# Real-world immune dynamics following COVID-19 vaccination and breakthrough infection: a paired-sample study in Zhejiang Province, China

**DOI:** 10.3389/fimmu.2026.1801564

**Published:** 2026-03-31

**Authors:** Yuxin Hu, Youhong Weng, Xiaodong Li, Yihua Huang, Jinren Pan, Yahan Wu, Rongxian Liao, Ruoyao Gong, Longyou Zhao, Dingmei Zhang, Mengke Han, Yu Xu, Xiaoliang Zheng, Shaohong Lu, Qingming Kong, Enfu Chen

**Affiliations:** 1 School of Laboratory Medicine and Bioengineering, Zhejiang Provincial People’s Hospital, Affiliated People’s Hospital, Hangzhou Medical College, Hangzhou, China; 2 Key Laboratory of Biomarkers and In Vitro Diagnosis Translation of Zhejiang Province, Zhejiang Provincial People’s Hospital, Affiliated People’s Hospital, Hangzhou Medical College, Hangzhou, China; 3 Department of Clinic Laboratory, The Fifth Affiliated Hospital of Wenzhou Medical University, Lishui, China; 4 Department of Clinic, Lishui Second People’s Hospital Affiliated to Wenzhou Medical University, Lishui, China; 5 Department of Infectious Diseases and Zhejiang Key Lab of Vaccine, Infectious Disease Prevention and Control, Zhejiang Provincial Center for Disease Control and Prevention, Hangzhou, China; 6 The Third Hospital of Quzhou, Quzhou, China; 7 Epidemiology Department, School of Public Health, Sun Yat-sen University, Guangzhou, China; 8 Zhejiang Key Laboratory of High-level Biosafety and Biomedical Transformation, Zhejiang Provincial People’s Hospital, Affiliated People’s Hospital, Hangzhou Medical College, Hangzhou, China; 9 Engineering Research Center of Novel Vaccine of Zhejiang Province, Zhejiang Provincial People’s Hospital, Affiliated People’s Hospital, Hangzhou Medical College, Hangzhou, China

**Keywords:** breakthrough infection, COVID-19, hybrid immunity, immune heterogeneity, lower-quartile immune response

## Abstract

**Introduction:**

As SARS-CoV-2 variants continue to spread, breakthrough infections (BTI) provide real-world insights into the durability and heterogeneity of COVID-19 vaccine-induced immunity. This study aimed to characterize the immune response dynamics and reconstruction characteristics after BTI in participants with different vaccination regimens in Zhejiang Province, China.

**Methods:**

A total of 370 participants were enrolled from a stratified random sample of permanent residents. Participants were either unvaccinated or had received one of the following vaccine regimens: one dose of adenoviral vector vaccine, two or three doses of inactivated vaccine, or, three doses of recombinant protein subunit vaccine. Paired serum samples were collected after vaccination and after BTI. RBD-IgG levels, pseudovirus neutralizing activity, and eight cytokines were systematically quantified. Correlation analyses and post-infection lower-quartile subgroup (T2-LQ) analyses were performed to assess immune heterogeneity.

**Results:**

All vaccine groups exhibited robust seroconversion, with post−vaccination RBD−IgG positivity exceeding 90% for both inactivated and recombinant vaccines. Following BTI, antibody levels and neutralizing activity increased significantly across all vaccine groups, consistent with the boosting effect of hybrid immunity. Cytokine profiling analyses revealed minimal differences between vaccine groups post-infection, with immune dynamics primarily driven by longitudinal changes. Notably, IL-13 levels declined consistently across all groups, accompanied by modest changes in the overall cytokine profile. Correlation analyses did not identify a stable concordance between antibodies and cytokines. However, variant-specific RBD-IgG measurements were highly correlated at both time points. Additionally, a post-infection lower-quartile subgroup (T2-LQ) was identified. Among BTI participants, baseline WT-specific RBD-IgG levels were comparable between T2-LQ and non-LQ individuals, suggesting that the low-responder phenotype is not simply attributable to pre-infection antibody levels.

**Conclusion:**

This paired−sample, population−based study suggests that BTI may further enhance humoral responses and be accompanied by changes in inflammatory/immunoregulatory markers on top of prior vaccine-induced immunity, while reducing some overall immune differences across vaccine platforms. The identification of the T2-LQ subgroup highlights persistent heterogeneity in post-infection immune responses and suggests the potential value of continued immune monitoring in the post-pandemic era.

## Background

Since the global pandemic of novel Coronavirus pneumonia (COVID-19) in 2019, the pandemic has continued to have a profound impact on the global public health system and socio-economic operations (1). According to the World Health Organization, the cumulative number of confirmed cases worldwide has exceeded 770 million, with more than 7 million direct deaths (2). In this context, large-scale vaccination, as a core prevention and control strategy for emerging infectious diseases, has become a key intervention to contain the spread of the epidemic and rebuild the herd immunity barrier (3–5). Vaccination strategies differ globally: while North America and Europe have widely adopted mRNA-based vaccines (e.g., BNT162b2, mRNA-1273), countries such as China have primarily utilized inactivated (e.g., CoronaVac), recombinant protein (e.g., ZF2001), and adenoviral vector (e.g., Convidecia) vaccines. These platforms differ significantly in immunogenicity, durability, and breadth of protection. The Omicron variant and its subline ages such as BA.5, has markedly increased viral transmissibility and immune evasion, leading to a rising incidence of breakthrough infection (BTI) among vaccinated individuals.

Compared with simple vaccination or natural infection, BTI provides a secondary antigenic stimulus on the basis of pre-existing vaccine-induced immunity, thereby eliciting a more complex and multi-layered immune response (6). Regarding humoral immunity, BTI can rapidly activate and expand the memory B cell population, promote its differentiation into plasma cells, thereby significantly increasing the neutralizing antibody titers and expanding the range of epitopes recognized by antibodies (7). Several studies indicate that this response can enhance cross-neutralization against multiple SARS-CoV-2 strains and variants (8). With respect to cellular immunity, BTI simultaneously enhances the helper functions of CD4^+^T cells and the cytotoxic activity of CD8^+^T cells, while also improving the multifunctionality and durability of the immune response (9). Hybrid immunity, resulting from both vaccination and natural infection, is associated with enhanced antibody titers, as suggested by recent studies indicating that heterologous vaccination strategies may lead to advantages in terms of antibody titers, breadth of immune recognition, and the durability of cellular immunity (10).

Importantly, BTI may trigger “immune remodeling,” reshaping the humoral and cellular immune architecture established by prior vaccination (11–13). This may promote convergence of immune profiles across different vaccine platforms. However, such remodeling may not fully compensate for pre-existing immune deficiencies, particularly in low-responder subgroups (14, 15).

The magnitude and longevity of the hybrid immune response can be influenced by multiple factors, including vaccine platform, number of doses, and the interval between BTI and the most recent vaccination (16). At present, longitudinal paired studies in real-world populations are still relatively limited, and their protective efficacy under different immune pathways and virus mutation backgrounds needs to be further verified (17).

In this study, we employed a multi-stage, stratified population-based and cluster random sampling approach, selecting 50 districts across 11 prefectural cities in Zhejiang province. From November 16, 2021 to April 5, 2023, serum samples were collected from all eligible participants to assess the level of neutralizing antibodies (NAbs), virus-specific cellular immune-related cytokines, and cross-neutralization activity. This investigation leveraged the diverse vaccine regimens deployed across Zhejiang and aimed to characterize post-BTI immune responses in a real-world population. Notably, we examined a lower-quartile subgroup (T2-LQ)-defined as individuals exhibiting immune responses in the bottom 25% of the post-infection distribution-to delineate features of weak or suboptimal immune reactivity. Such low-responder phenotypes are critical to identify in the post-pandemic era, as they may reflect incomplete immune protection even in individuals with hybrid exposure histories. These findings highlight persistent heterogeneity in post-vaccination and post-infection immunity and underscore the importance of continued immune monitoring in populations with hybrid immunity.

## Materials and methods

### Study design and participants

This retrospective, population-based study investigated the immune responses induced by different COVID-19 vaccination regimens and subsequent breakthrough infections (BTIs) among healthy individuals in Zhejiang Province, China. A multi-stage, stratified cluster random sampling method was adopted to ensure broad representativeness. In the first stage, 50 districts were randomly selected from 11 prefecture-level cities across the province. In the second stage, residents within each district were stratified by age (<20 years, 20–60 years, >60 years) and sex (male, female), resulting in six demographic strata. Random sampling was performed within each stratum to ensure balanced enrollment. Participants were categorized into five groups based on vaccination history: CV-2 (n=114): Two doses of inactivated vaccine (CoronaVac; Sinovac Biotech); CV-3 (n=47): Three doses of inactivated vaccine (CoronaVac; Sinovac Biotech); RP-3 (n=93): Three doses of recombinant protein subunit vaccine (ZF2001; Anhui Zhifei Longcom, CHO cell-based); RA-1 (n=31): One dose of adenovirus vector vaccine (Convidecia; CanSinoBIO); C-0 (n=85): Unvaccinated individuals serving as baseline controls. All vaccination histories reflected participants’ real-world vaccination records obtained through the routine public vaccination program prior to enrollment. Serum samples were collected at two time points: T1 (post-vaccination baseline): November 16, 2021 to January 2022; T2 (post-BTI follow-up): March 28 to April 5, 2023. BTIs occurred during the major epidemic wave in December 2022-January 2023. Based on contemporaneous genomic surveillance, BA.5.2- and BF.7-related Omicron lineages predominated in China and Zhejiang during this period (18). Paired T1/T2 samples (from the same individuals) accounted for 35.95% (133/370) of the final analytic cohort, while the remaining participants contributed single time-point samples. All participants were tested for anti–SARS-CoV-2 RBD-specific IgG antibody levels, pseudovirus neutralizing activity (IC_50_), and eight serum cytokines (IL-2, IL-4, IL-5, IL-6, IL-10, IL-13, IFN-γ, and TNF-α). All BTI cases included at T2 had been confirmed by RT-PCR diagnosis at certified medical institutions across Zhejiang Province. Ethical approval for the study was granted by the Ethics Committee of the Zhejiang Provincial Center for Disease Control and Prevention (Approval No. 2020-045-01).

### Serum isolation

Whole blood samples (3mL) were collected from each participant via venipuncture into serum separation tubes. Samples were allowed to clot at room temperature for 30–60 minutes and were subsequently and centrifuged at 3500 rpm for 15 minutes to isolate serum. For biosafety purposes, all serum samples were inactivated at 56 °C for 30 minutes prior to downstream assays. Inactivated sera were aliquoted (500 µL per vial) and then stored at -20 °C until batch testing.

### RBD-IgG Ab concentration assays

IgG antibodies targeting the receptor-binding domain (RBD) of the SARS-CoV-2 prototype strain (WT), Delta (L452R, T478K), and Omicron BA.5 variants were measured using an indirect enzyme-linked immunosorbent assay (iELISA). Briefly, RBD antigens (Yiqiao Shenzhou Biotechnology Co., Ltd., Beijing, China) were coated onto 96-well plates (NEST, 504201) at 5 µg/mL in 0.05 M Na_2_CO_3_-NaHCO_3_buffer (pH 9.6) overnight at 4 °C. Following PBST washes (PBS with 0.05% Tween-20, pH 7.4), 96-well plate were incubated with 5% skim milk in PBST (250 μL/well) for 2 hours at 37 °C to block nonspecific binding, then washed again. Serum samples were diluted 1:10 in blocking buffer and incubated at 100 µL/well for 1 h at 37 °C. After washing, HRP-conjugated goat anti-human IgG (Solarbio, Beijing, China) was diluted 1:5000 in PBST with 5% skim milk, added at 100 µL per well, incubated for 1 h at 37 °C, and then washed. TMB substrate solution (100 μL/well) was added and incubated at 37 °C for 10–15 minutes in the dark, after which 50 μL/well of 2 M sulfuric acid was added to stop the reaction. Absorbance was measured at 450 nm using a BioLegend ELISA Mini Plate Reader. The reference standard was prepared by mixing samples with high serum antibody concentration and a standard curve was plotted after dilution. The initial concentration was set to 20,000 (unit/mL).

### Cytokine assays

Serum concentrations of cytokines IL-2, IL-4, IL-5, IL-6, IL-10, IL-13, IFN-γ, and TNF-α were quantified using the LEGENDplex™ multiplex flow cytometry cytokine detection kit (Shanghai Xing jianna Biotechnology Co., Ltd.). A 2× working dilution of the inactivated serum, assay buffer, and cytokine mixture was prepared at a 1:1:1 ratio and added to a 96-well plate (50 µL/well). After incubation with gentle shaking at 37 °C for 2 hours, the plate was centrifuged at 350 × g for 5 minutes, and the supernatant was immediately discarded. Detection antibodies (25 µL/well) were then added, and the plate was incubated in the dark with shaking at 37 °C for 1 hour. Subsequently, SA-PE (25 µL/well) was added directly, followed by a 30-minute incubation with shaking at 37 °C. After centrifugation at 1000 × g for 5 minutes, the supernatant was immediately discarded. The beads were washed once with 1× wash buffer, then resuspended in 200 µL of 1× wash buffer per well and vortexed for 1 minute. Finally, 200 µL of the bead suspension was transferred to a new Eppendorf tube for analysis on a flow cytometer. Standard curves were generated using kit-provided standards, ranging from 10,000 pg/mL to serial dilutions. Each 96-well plate included its own standard curve to allow plate-specific calibration and reduce inter-plate variability.

### Pseudovirus-neutralizing activity assays

The pseudovirus neutralization assay was performed according to our previously published methods (19). Two pseudovirus strains, PSV001 (WT) and PSV023 (Omicron BA.5), were used to represent the ancestral SARS-CoV-2 strain and the Omicron BA.5 variant, respectively, for assessing the neutralizing activity of serum antibodies. Serum samples were subjected to 2-fold serial dilutions, and each dilution was tested in duplicate. Briefly, 50 μL of diluted serum was mixed with 50 μL of pseudovirus working solution (WT or BA.5) and incubated at 37 °C for 1 h, followed by the addition of 293T/ACE2-TMPRSS2 cells (30,000 cells per well, 96-well plate). Controls were set as follows: 50µL of basal DMEM mixed with 50 µL of pseudovirus served as the virus control (positive control), and 100 µL of complete DMEM without pseudovirus served as the cell background control (negative control). The reference antibody had an initial concentration of 2,280 µg/mL and was diluted 1:5 before use. After incubation for 72 h at 37 °C with 5% °CO_2_, luminescence (RLU) was measured using a chemiluminescence plate reader to quantify luciferase activity. The inhibition rate was calculated as follows: Inhibition (%) = 1- (Mean RLU sample - Mean RLU negative control)/(Mean RLU positive control - Mean RLU negative control).

IC50 values were determined using the Reed-Muench method. Samples that did not reach 50% inhibition at the lowest dilution (1:16), and therefore had no calculable IC50, were assigned a value of 0 (undetectable).

### Statistical analysis

Statistical analyses were performed in R (v4.4.1). Group differences were assessed using linear mixed-effects models, including vaccine group and timepoint (and their interaction where applicable) as fixed effects and a subject-specific random intercept. Pairwise comparisons between groups were based on model-estimated marginal means with Tukey adjustment for multiple comparisons. For analyses involving multiple immune features tested in parallel, *P* values were adjusted using the Benjamini-Hochberg false discovery rate (BH-FDR). For endpoint variables containing zero values, a log10(X + c) transformation was applied to enable linear model analyses. Key comparisons were repeated using different c values as a sensitivity analysis, and the main conclusions remained consistent across a reasonable range of c values (**Supplementary Figure 1**). All tests were two-sided, and an adjusted *P* value (or *q* value) < 0.05 was considered statistically significant. Statistical significance is indicated as follows: *p < 0.05, **p < 0.01, ***p < 0.001, ****p < 0.0001.

An adjusted composite immune index was calculated from WT-, Delta-, and BA.5-specific anti-RBD IgG levels and eight cytokines. For each feature, log10-transformed values were adjusted for vaccine group, age, sex, and Days_T1_T2. Adjusted values were converted to percentile ranks across participants, and the mean percentile rank across all 11 features was used as the adjusted immune index. T2-LQ was defined as the lowest quartile of this index. For visualization, humoral and cytokine subscores were calculated as the mean Z-scores of the three anti-RBD markers and eight cytokines, respectively.

## Result

### Demographic characteristics of the participants

**Figure 1:**
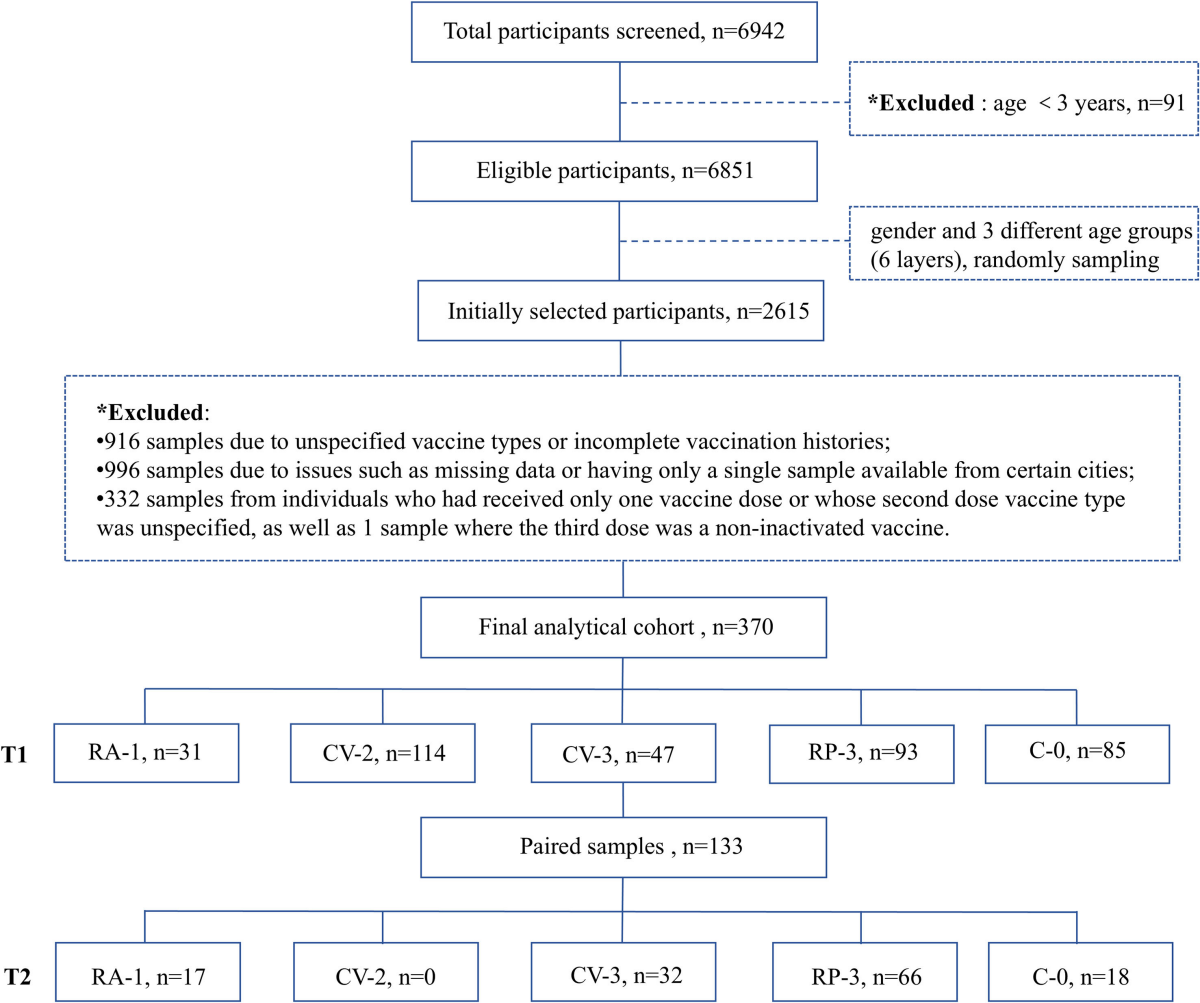
Study design and participant flowchart with paired and cross-sectional sampling. Flowchart illustrating participant inclusion and exclusion, vaccination group allocation, and serum sampling at T1 (post-vaccination) and T2 (post-breakthrough infection). Of the 370 participants included in the final analyses, 133 (35.95%) contributed paired samples at both time points, while the remaining participants contributed valid data at a single time point. Vaccine groups included RA-1 (one dose of adenoviral vector vaccine), CV-2 (two doses of inactivated vaccine), CV-3 (three doses of inactivated vaccine), RP-3(three doses of recombinant protein vaccine), and C-0 (unvaccinated group).

A flow chart with the participants and subgroups included in the analyses (**Figure 1**). From an initial sample pool of 6,942 individuals, 91 infants under the age of 3 were excluded, leaving 6,851 eligible individuals aged ≥3 years. The remaining population was stratified by sex (male/female) and age (<20 years, 20–60 years, >60 years), and random sampling was performed within each stratum to obtain a study sample with approximately balanced sizes across the six strata. To preserve adequate representation in each stratum after data cleaning, an initially larger stratified sample was selected. After sampling, participants were excluded if they had missing samples, only one available sample, unclear or incomplete vaccination timelines, or vaccination histories inconsistent with the predefined study groups, participants were then assigned to the RA-1, CV-2, CV-3, RP-3, or unvaccinated control (C-0) cohorts based on immunization history. In total, 370 participants were enrolled and the demographic characteristics of the vaccinated population, detailing the distribution across regions and the various vaccine immunization schedules administered is outlined in **Table 1**. Of the participants,180 (48.65%) were male and 190 (51.35%) were female, with a median age of 50 years. Among the different vaccine types, the adenovirus vector vaccine had the lowest representation (8.38%) (**Supplementary Table 1**). In contrast, the inactivated vaccine was the most commonly administered regimen (43.51%). Among them, 114 individuals (30.81%) received two doses, while 47 individuals (12.70%) completed a three-dose schedule. Among participants with paired sera included in the longitudinal analysis, baseline characteristics and sampling intervals are summarized in **Supplementary Table 2**. Group-wise comparisons of the interval from the most recent vaccine dose to T1 sampling are provided in **Supplementary Table 3**.

**Table 1:** Characteristics of 370 vaccinators.

Characteristic	Group	Number of people	Constituent ratio
Total		370	100.00%
Sex	Male	180	48.65%
	Female	190	51.35%
Age	3-19	72	19.46%
	20-39	77	20.81%
	40-59	84	22.70%
	≥60	137	37.03%
Vaccine types	Unvaccinated	85	22.97%
	Inactivated vaccine (2 doses)	114	30.81%
	Inactivated vaccine (3 doses)	47	12.70%
	Recombinant subunit vaccine (3 doses)	93	25.14%
	Adenovirus vector vaccine (1 doses)	31	8.38%
Area	Hangzhou	45	12.16%
	Ningbo	60	16.22%
	Wenzhou	27	7.30%
	Huzhou	36	9.73%
	Jiaxing	3	0.81%
	Shaoxing	35	9.46%
	Jinhua	48	12.97%
	Quzhou	31	8.38%
	Zhoushan	24	6.49%
	Taizhou	37	10.00%
	Lishui	24	6.49%

### Humoral antibody responses following vaccination and breakthrough infection

**Figure 2:**
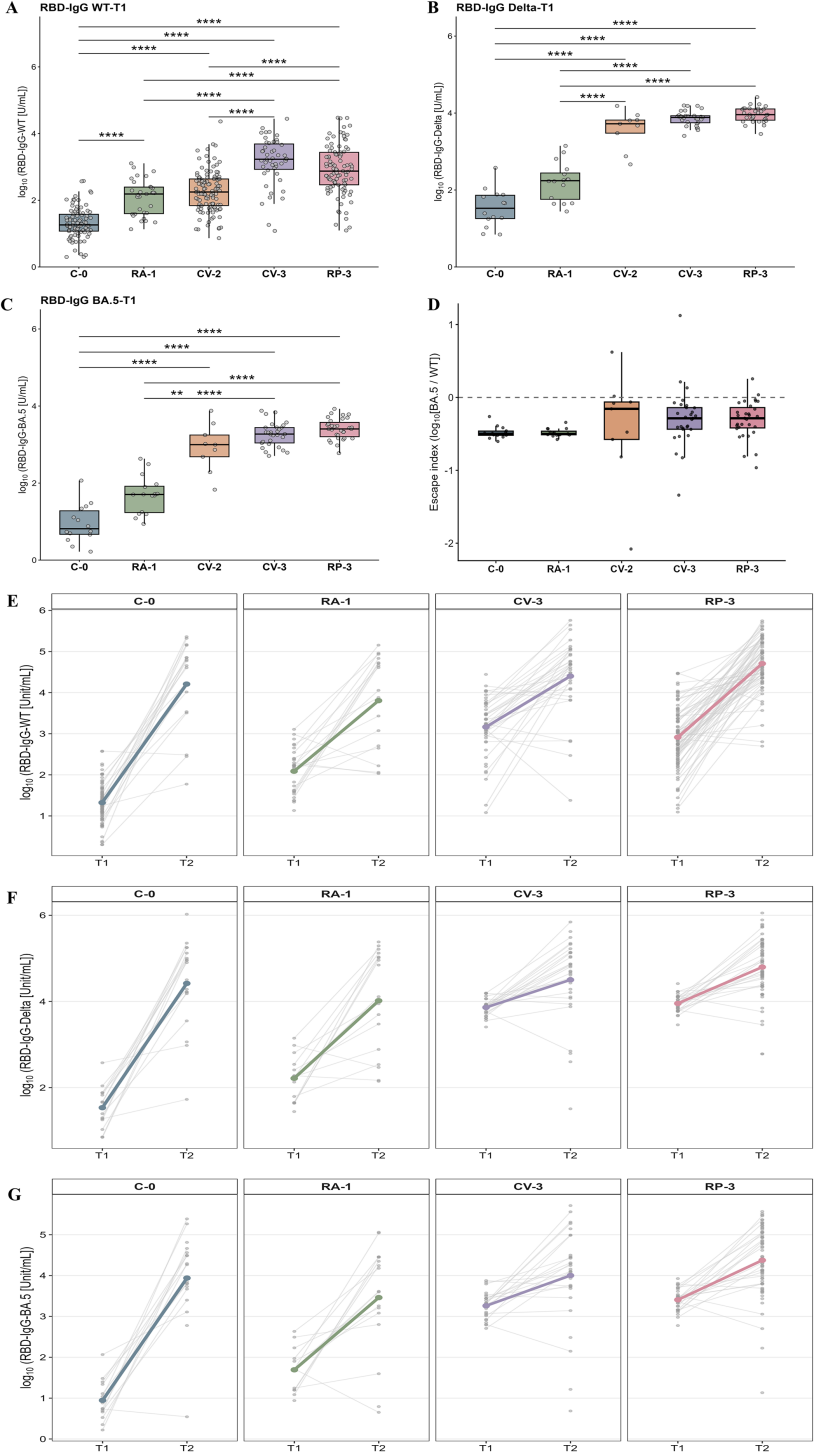
Variant-specific humoral responses and immune escape following vaccination and breakthrough infection. **(A-C)** anti-RBD IgG levels against wild-type (WT), Delta, and Omicron BA.5 at T1 (post-vaccination), shown on the log10 scale. Pairwise between-group comparisons were performed using linear mixed-effects models with Tukey adjustment. (**P < 0.01, ****P < 0.0001). At T1, WT RBD-IgG was measured in the full cohort (N = 370), whereas Delta- and BA.5-RBD IgG were measured only in a predefined subset with relatively high WT RBD-IgG levels (N = 99). **(D)** BA.5 escape index at T1, defined as the within-participant log10 difference between BA.5- and WT-specific anti-RBD IgG levels (log_10_[BA.5] − log_10_[WT]); more negative values indicate greater immune escape relative to WT. The escape index is a relative metric (BA.5/WT) and can remain negative even when absolute IgG levels increase; absolute T1–T2 changes are quantified in paired samples using longitudinal mixed-effects models (**Supplementary Table 6**). **(E-G)** Longitudinal changes in variant-specific anti-RBD IgG levels from T1 to T2 among participants with paired samples. **(E)** WT-specific anti-RBD IgG in all participants with paired WT measurements (N = 133). **(F, G)** Delta- and BA.5-specific anti-RBD IgG in the overlapping paired subset with measurements available at both T1 and T2 for the respective variant (total n = 69; C-0, n = 14; RA-1, n = 14; CV-3, n = 20; RP-3, n =21). Longitudinal inference aligned with the repeated-measures structure is provided in **Supplementary Tables 5**, **6**.

To comprehensively evaluate the humoral immune response after different vaccination regimens and subsequent BTI in Zhejiang Province, we quantified serum anti-RBD IgG antibodies levels. In the absence of pre-pandemic negative sera, we derived an operational seropositivity cutoff by discriminant analysis of the unvaccinated group (C-0, n = 85) versus post-vaccination samples (n = 285). ROC analysis showed high classification performance (AUC = 0.9239, 95% CI 0.8961-0.9517; *P* < 0.0001), with a maximum Youden index of 0.7201, yielding a cutoff of 106.9 U/mL. Five unvaccinated samples exceeded this threshold, consistent with unrecognized prior infection or background reactivity, and age-stratified seropositivity rates are provided in **Supplementary Table 4**. At T1, seropositivity exceeded 90% in CV-3 and RP-3; after infection, seropositivity increased across all groups and reached 100% in RA-1 and RP-3 among BTI cases. Quantitatively, anti-RBD IgG levels at T1 were higher in all vaccinated groups than in C-0, with the highest levels observed in CV-3 and RP-3 (**Figure 2A**). After BTI, RP-3 remained higher than RA-1 (**Supplementary Figure 2A**).

We measured IgG binding to WT, Delta-RBD (L452R and T478K), and Omicron BA.5 RBD antigens in a subset with higher anti-RBD IgG levels (n = 99) to assess cross-variant reactivity. At T1, the CV-2, CV-3 and RP-3 groups showed significantly higher IgG levels against both Delta–RBD and BA.5-RBD than the C-0 and RA-1 groups (**Figures 2B, C**). Among participants with BTI, variant-specific IgG responses were evaluated in the overall paired BTI cohort (n = 133). In this cohort-wide post-BTI analysis, only the RP-3 group showed significantly higher BA.5-specific IgG levels than the RA-1 group (**Supplementary Figures 2B, C**). Given the different analytical populations at T1 and T2, the cross-sectional findings mainly describe the immune profiles at each time point. After adjustment for age and sex, vaccine group remained independently associated with antibody levels. This association remained materially unchanged in time-adjusted sensitivity analyses (**Supplementary Table 5**), supporting differences in humoral response magnitude across immunization regimens.

To further assess immune escape patterns of SARS-CoV-2 variants, we calculated a BA.5 escape index, defined as the within-individual log_10_ ratio of BA.5- to WT-specific RBD-IgG levels: Escape index = log_10_[BA.5/WT]. This index quantifies the degree of antigenic divergence and immune evasion by the Omicron BA.5 variant. Following vaccination, the BA.5 escape index was predominantly negative across vaccine groups, indicating a generally reduced BA.5-specific RBD binding antibody response relative to WT (**Figure 2D**). In contrast, the Delta escape index showed an overall positive distribution (**Supplementary Figure 2D**), consistent with a comparatively smaller reduction in Delta-specific binding relative to WT. Compared to Delta, BA.5 exhibited significantly lower IgG recognition across all vaccine groups (*P* < 0.0001), consistent with its known immune evasiveness and multiple spike protein mutations within the RBD domain. These results highlight reduced cross-variant binding recognition of vaccine-induced antibodies to highly divergent Omicron sublineages. We further evaluated the BA.5 escape index after natural infection (T2) in groups with paired samples (C-0, RA-1, CV-3, and RP-3), and values remained mostly below zero (**Supplementary Figure 2E**), suggesting that reduced relative recognition of BA.5 persisted after infection despite overall boosting of antibody levels.

Longitudinal analyses of the groups with paired samples at both time points (C-0, RA-1, CV-3 and RP-3) showed that anti-RBD IgG levels against WT, Delta -RBD and BA.5-RBD increased from T1 to T2, although the magnitude of increase differed across regimens (**Figures 2E–G**); CV-2 was excluded because no T2 samples were available following a third dose). After additional adjustment for key timing variables in sensitivity analyses (the vaccination-to-T1 interval and the T1–T2 sampling interval), between-group differences remained (**Supplementary Table 6**).

To assess functional humoral neutralization, we measured pseudovirus neutralizing activity and quantified it as the 50% inhibitory dilution (IC50). Because low-binding sera frequently yield IC50 values below the assay LLOD, IC50 was not measured in the full cohort but in an enriched higher WT RBD-IgG subset within each vaccine group. IC50 values were analyzed on a log scale after handling measurements at/below the assay detection limit (**Supplementary Methods**). After vaccination, IC50 was generally higher in the inactivated and recombinant protein vaccine groups; in contrast, a substantial proportion of samples in C-0 and RA-1 had IC50 values at or near the assay detection limit (**Supplementary Figure 2F**). After natural infection, IC50 distributions shifted upward across groups and between-group differences were not apparent (**Supplementary Figure 2G**). In paired longitudinal samples, changes in IC50 were heterogeneous across individuals: CV-3 showed an overall downward trend, whereas the other groups tended to increase, consistent with enhanced neutralization after infection (**Supplementary Figure 2H**). In addition, anti-RBD IgG levels were positively correlated with IC50 (**Supplementary Figure 2I**). Notably, IC50 was measured only in a serum subset with higher anti-RBD IgG levels; thus, these results are intended to provide functional support and describe trends, and should not be extrapolated to the neutralization titers distribution of the full cohort or used for strict between-group inference.

Overall, antibody levels after vaccination were generally higher in the CV-3 and RP-3 groups, followed by RA-1 and CV-2. Among vaccinated participants who experienced breakthrough infection (BTI), antibody levels increased further at T2 relative to T1, suggesting that BTI can boost humoral immune responses.

### Cytokine profiling

To evaluate cellular immune responses following vaccination and BTI, we measured a variety of key cytokines in serum, including Th1-associated cytokines (IL-2, IFN-γ, TNF-α) and Th2-associated cytokines (IL-4, IL-5, IL-6, IL-10, and IL-13).

**Figure 3:**
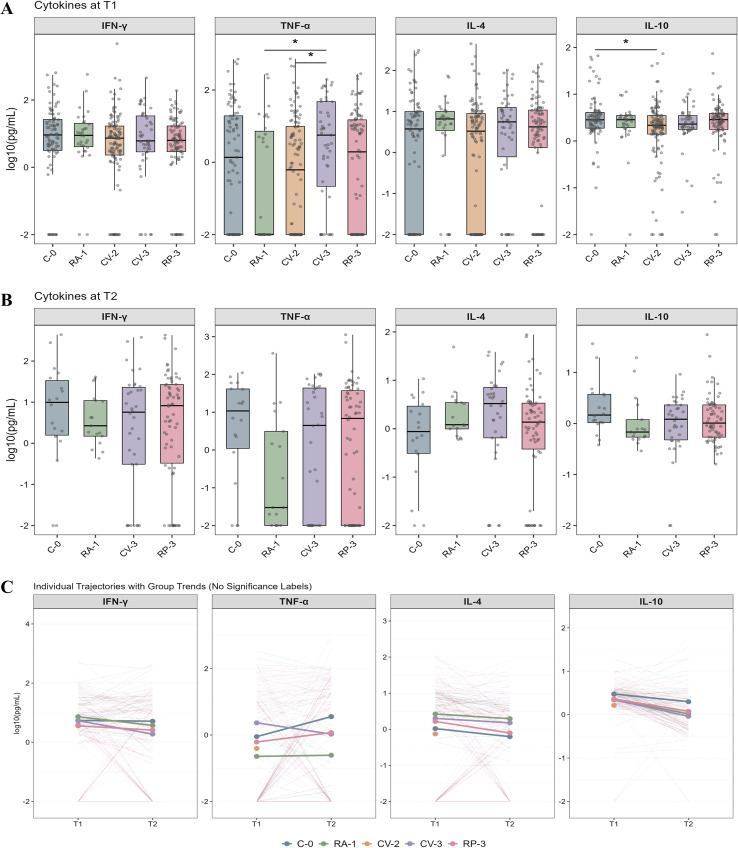
Serum cytokine dynamics before and after breakthrough infection across vaccine platforms. **(A)** Distributions of selected cytokines at T1 (post-vaccination) across vaccine group. **(B)** Distributions of selected cytokines at T2 (post-infection). **(C)** Within-participant longitudinal trajectories from T1 to T2, illustrating cytokine dynamics post infection. Cytokine levels are shown on the log10 scale and analyzed using linear mixed-effects models with adjustment for age, sex, and repeated measures. (Handling of values at/below the detection limit and sensitivity analyses are provided in **Supplementary Figure 1**. *P < 0.05). Cross-sectional between-group inference was based on linear mixed-effects models with Tukey-adjusted pairwise comparisons within each time point; longitudinal inference accounted for the repeated-measures structure (**Supplementary Table 9**). Sample sizes for each panel are provided in **Supplementary Table 10**.

At T1 (post-vaccination), cytokine levels were compared using age- and sex-adjusted models. In the Th1-related cytokine profile, TNF-α was higher in the CV-3 group than in both the CV-2 and RA-1 groups (*P* = 0.029, *P* = 0.032), whereas no significant differences were observed for IFN-γ. A significant pairwise difference was also observed for IL-2, with higher levels in the RP-3 group than in the CV-2 group (*P* = 0.045) (**Figure 3A**; **Supplementary Figure S3A**). In the Th2-related-inflammatory cytokine profile, IL-10 was lower in the two-dose inactivated vaccine group than in the unvaccinated group (*P* = 0.026). No significant differences were observed in the other cytokines (IL-4, IL-5, IL-6 and IL-13) among the groups. (**Figure 3A**; **Supplementary Figure S3A**). To assess potential confounding by vaccination timing, we performed a vaccinated-only sensitivity analysis. After additional adjustment for Days_Since_Vax, the TNF-α difference was attenuated and no longer statistically significant (**Supplementary Tables S7**, **8**). Following BTI, no significant differences in cytokine levels were observed among vaccine groups (**Figure 3B**; **Supplementary Figure S3B**).

Paired longitudinal analyses demonstrated that cytokine changes from T1 to T2 were heterogeneous across analytes and vaccine groups (**Figure 3C**; **Supplementary Figure 3C**). Among Th1-related cytokines, IL-2 and TNF-α increased significantly only in the C-0 group, whereas IFN-γ showed no significant within-group longitudinal change after BH-FDR correction. By comparison, Th2-related cytokines exhibited more consistent longitudinal patterns after BTI. IL-13 decreased consistently and significantly in all groups, representing the most stable longitudinal signal in this study. In addition, IL-5 decreased in the C-0, CV-3 and RP-3 groups, whereas IL-4 showed a significant decline only in the RP-3 group. Beyond classical Th2 cytokines, IL-10 decreased significantly in the RA-1, CV-3, and RP-3 groups, and IL-6 showed a similar decline in these same groups. These findings indicate that BTI convalescence was accompanied by limited longitudinal cytokine changes, with Th2-related changes, particularly the consistent decline in IL-13, being the most reproducible pattern. All inferences were based on linear mixed-effects models with BH-FDR correction, and effect sizes (Δlog10[T2−T1]) and adjusted results are provided in **Supplementary Table 9**.

To further characterize cytokine profile features across immune backgrounds, we performed an exploratory principal component analysis (PCA) using log10(x+ 0.01)-transformed serum cytokine values, followed by centering and scaling (**Supplementary Figure 4**). PCA showed substantial overlap between T1 and T2, with only a modest shift after BTI rather than strong global separation (**Supplementary Figure 4A**). In paired samples, many individuals shifted from T1 to T2, but the trajectories were heterogeneous (**Supplementary Figure 4B**). The loading plot showed that PC1 reflected a shared cytokine axis, whereas PC2 captured relative differences among cytokine subsets (**Supplementary Figure 4C**). These results support a slight shift, rather than strong reorganization, of the serum cytokine profile after BTI. As a complementary descriptive analysis, we also visualized relative cytokine levels across groups using heatmaps (**Supplementary Figure 4D**; **Supplementary Table 10**). Notably, serum cytokines reflect an integrated systemic immune signal and cannot be directly equated with Th1/Th2 polarization of antigen-specific T cells. Therefore, we interpret these findings as phenotypic clues of immune milieu changes during post-infection convalescence, rather than as mechanistic evidence of T helper subset switching.

Overall, the cytokine results showed limited between-group differences after vaccination, and these differences became even less apparent after BTI. In contrast, longitudinal changes after BTI were modest but more consistent for Th2-/inflammation-related cytokines than for Th1-associated cytokines, with IL-13 showing the clearest and most reproducible decline across all groups.

### Correlations and integrative analysis of immune-response parameters

Antigen exposure quality is shaped not only by the magnitude of individual immune components but also by their coordination. Given their distinct mechanisms of action, coordinated responses across multiple immune layers may better reflect an individual’s overall immune state and potential protective capacity than any single immune parameter alone.

Accordingly, we examined covariate-adjusted associations among immune-response parameters to assess whether humoral responses systematically covary with inflammatory and immunoregulatory factors. We assessed the internal consistency of variant-specific RBD-IgG at T1 and T2. WT-, Delta-, and BA.5-specific RBD-IgG levels were highly correlated at both time points. Therefore, we derived a composite RBD response score (RBD_score) to capture overall humoral responsiveness for subsequent integrative analyses (**Supplementary Figure 5**).

**Figure 4:**
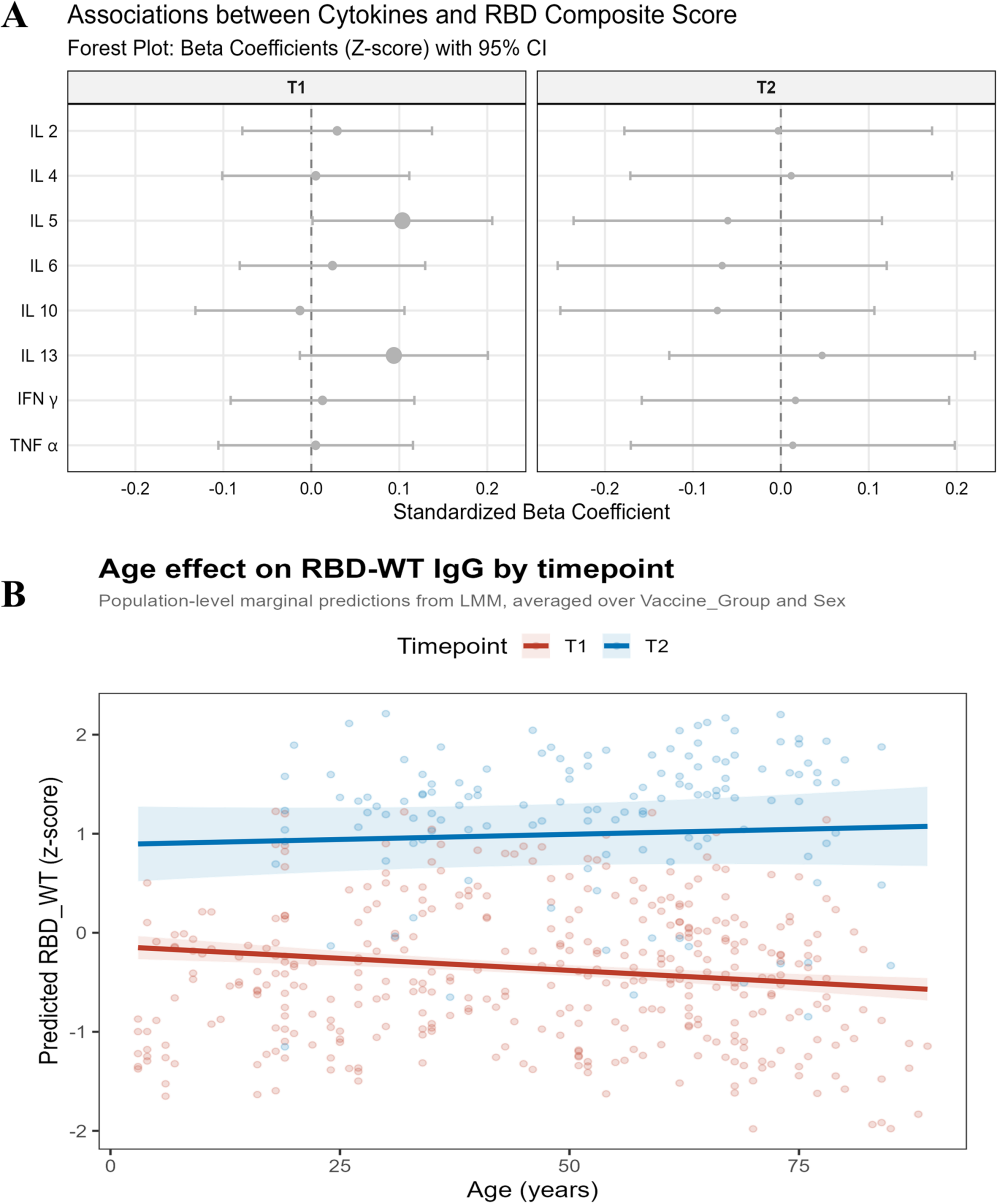
Covariate-adjusted associations between humoral and cytokine immune responses. **(A)** Forest plots showing standardized regression coefficients (β) for associations between a composite RBD-IgG response score and individual cytokines at T1 and T2, adjusted for age, sex, vaccine group, and timing covariates from multivariable linear models with 95% confidence intervals. Multiple testing within each time point across eight cytokines was controlled using the Benjamini-Hochberg FDR procedure (m = 8). Full statistical results and sample sizes for each panel are provided in **Supplementary Table 11**. **(B)** In the mixed-effects model, a significant Age × Timepoint interaction was observed (*P* = 0.024). At T1, WT RBD-IgG decreased significantly with age (*P* < 0.001), whereas at T2 the age association was attenuated and no longer statistically significant (*P* = 0.468). The plotted lines represent population-level marginal predictions averaged over vaccine group and sex, and shaded areas indicate 95% confidence intervals.

We then applied multivariable linear models to estimate covariate-adjusted associations between the RBD_score and eight serum cytokines, and summarized effect sizes with their uncertainty in forest plots (**Figure 4A**). After BH-FDR (m = 8/time point), no associations remained statistically significant (all *q* > 0.05), indicating that within the sampling window of this study, serum cytokine levels had limited explanatory power for inter-individual variation in humoral responses (**Supplementary Table 11**).

In addition, we explored host-related factors associated with humoral responses and observed a time point-dependent association between age and WT-specific anti-RBD IgG levels (*P* = 0.024). At T1, antibody levels decreased significantly with increasing age (*P* < 0.001), whereas this association was attenuated and no longer statistically significant at T2 (*P* = 0.468) (**Figure 4B**). These findings suggest that the age-related pattern of WT RBD-IgG levels may differ between the post-vaccination and post-breakthrough infection phases.

### Post-infection immune heterogeneity and the lower-quartile subgroup

**Figure 5:**
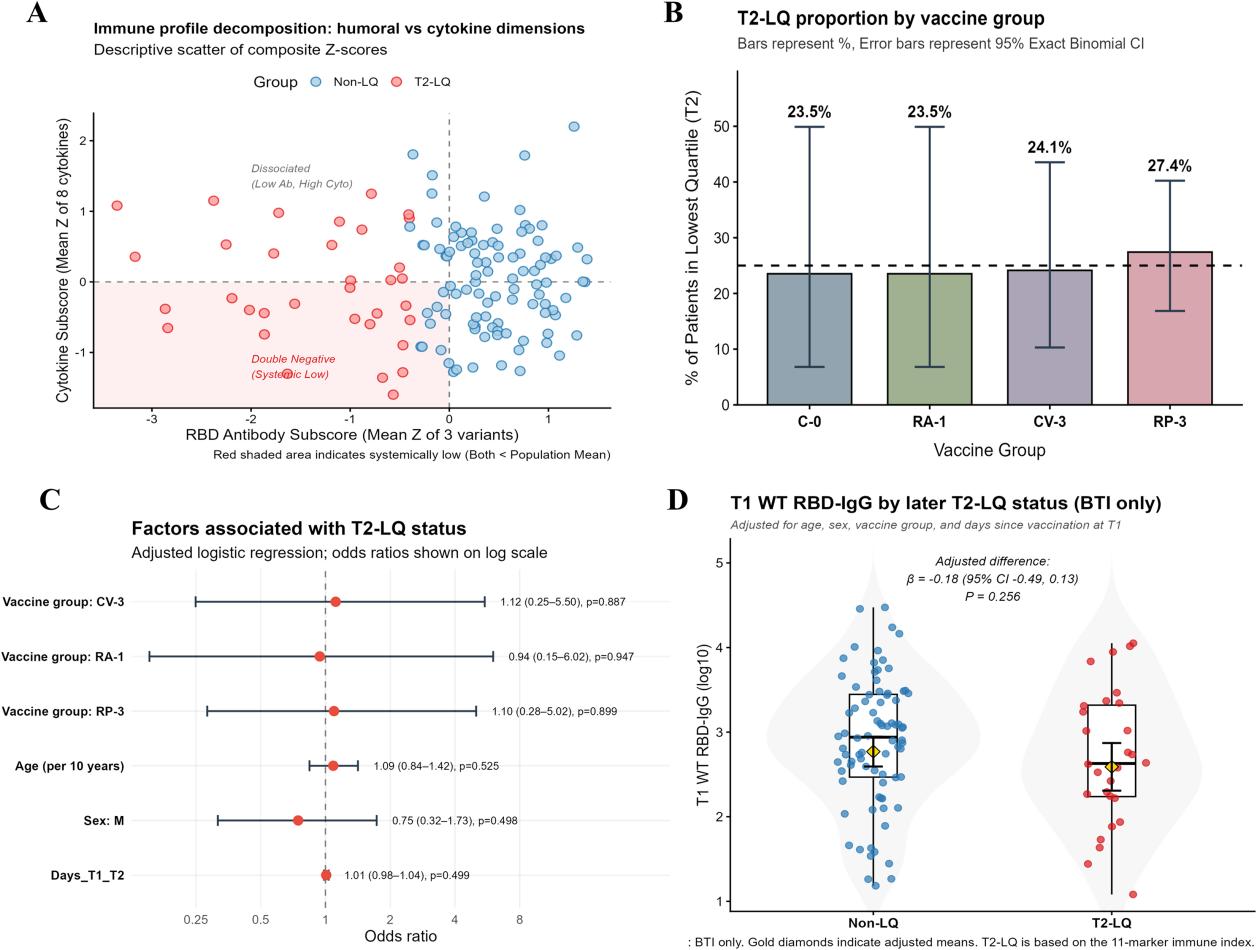
Identification and characteristics of the lower-quartile post-infection immune response group (T2-LQ). **(A)** Descriptive projection of T2-LQ and non-LQ participants onto humoral and cytokine dimensions. The x-axis shows the RBD antibody subscore, defined as the mean Z-score of WT-, Delta-, and BA.5-specific anti-RBD IgG. The y-axis shows the cytokine subscore, defined as the mean Z-score across eight cytokines. A value of 0 denotes the population mean; lower values indicate relatively lower levels. The red shaded area marks the descriptive dual-low region, where both subscores are below the population mean. T2-LQ status was defined using the adjusted immune index and is shown here only as an overlay for descriptive visualization. **(B)** Proportion of participants classified as T2-LQ across vaccine groups. Error bars indicate exact binomial 95% confidence intervals; the dashed line marks the overall 25% cutoff (lowest quartile). **(C)** Odds ratios (ORs) and 95% confidence intervals were estimated using adjusted logistic regression. T2-LQ was defined as the lowest quartile of the cohort-specific immune index at T2. The immune index was derived from 11 immune markers using covariate-adjusted residual percentile ranks. ORs should be interpreted as descriptive associations. **(D)** Comparison of pre-infection (T1) WT-specific RBD-IgG levels between T2-LQ and non-LQ participants among BTI cases. Adjusted differences (β, 95% CI) were estimated using multivariable linear models controlling for age, sex, vaccine group, and days since the most recent vaccination at T1; points represent individuals. Sample sizes were C-0 (n = 17), RA-1 (n = 17), CV-3 (n = 29), and RP-3 (n = 62). T2-LQ (n=32, about 25%).

To explore inter-individual heterogeneity in immune responses following vaccination and BTI, we first calculated an adjusted composite immune index at T2 using 11 immune features and defined the lowest quartile as the T2 lower-quartile subgroup (T2-LQ). We then projected T2-LQ and non-LQ participants onto a two-dimensional T2 immune landscape based on the RBD antibody subscore and cytokine subscore for descriptive visualization (**Figure 5**; **Supplementary Figure S6**).

The adjusted composite immune index exhibited a continuous distribution across the study population (**Supplementary Figure 6A**). Accordingly, rather than applying a predefined clinical protection threshold, we used a quantile-based approach and defined the lowest quartile as T2-LQ. T2-LQ classification remained highly consistent across alternative scoring strategies, including percentile ranking versus Z-score standardization (**Supplementary Figure 6B**), and when the feature set was reduced from the full panel of 11 immune metrics to a prespecified core subset of 7 metrics (**Supplementary Figure 6C**).

When visualized across the humoral and cytokine dimensions, T2-LQ individuals were more often located in regions with lower humoral subscores, with a subset also showing lower cytokine subscores, and tended to cluster toward the lower-left portion of the plot (**Figure 5A**; overall immune landscape in **Supplementary Figure 6D**).

With respect to group composition, the proportion of individuals classified as T2-LQ varied modestly across vaccine groups. Overall, the proportions were broadly similar, although the RP-3 group showed a numerically higher proportion of T2-LQ individuals than the other groups. However, the confidence intervals were wide and overlapping, indicating that these between-group differences should be interpreted as descriptive rather than statistically robust (**Figure 5B**). Consistently, multivariable logistic regression analyses did not identify clear associations between T2-LQ membership and vaccine group, BTI status, or demographic characteristics (**Figure 5C**, **Supplementary Figure S6E**). In addition, the distribution of the T1–T2 sampling interval was comparable between T2-LQ and non-LQ individuals (**Supplementary Figure 6F**), suggesting that low-tail classification was unlikely to be driven primarily by systematic differences in follow-up timing.

To further assess whether T2-LQ status simply reflected baseline humoral differences, we compared pre-infection (T1) WT-specific anti-RBD IgG levels within the BTI subset. T1 WT anti-RBD IgG levels were comparable between T2-LQ and non-LQ individuals, and this finding remained unchanged after additional adjustment for the interval between the most recent vaccination and T1 sampling (**Figure 5D**). Together, these results suggest that T2-LQ status was not explained solely by baseline antibody levels, but may reflect heterogeneity in post-infection immune responses beyond pre-infection humoral status.

Taken together, we identified a robust lower-quartile subgroup of post-infection immune responses, but this pattern was not readily explained by vaccination regimen, baseline demographics, or differences in follow-up timing. Notably, lower-quartile individuals did not exhibit uniformly reduced pre-infection humoral responses. The identification of such lower-quartile subgroup provides a framework for understanding heterogeneity in vaccine- and infection-induced immunity and lays the groundwork for future studies to evaluate its association with clinical outcomes and the potential need for targeted booster or individualized immunization strategies.

## Discussion

COVID-19 vaccination induces specific humoral and cellular immunity against SARS-CoV-2, effectively reducing the incidence of severe disease after infection (20). In this real-world paired-sample study, we systematically assessed antibody and cytokine responses following BTI among individuals who had received different vaccination regimens, including inactivated, recombinant subunit, and adenovirus-vectored vaccines. Using a comparative design and incorporating multiple immune measures, we characterized immune heterogeneity across vaccine platforms and further identified a post-infection lower-quartile subgroup. Together, these analyses describe immune remodeling and inter-individual variation in the context of hybrid immunity.

### i. Humoral response differences across regimens and overall boosting after BTI

In this study, antibody responses were detected in all participants. We observed a graded difference in post-vaccination anti-RBD IgG levels across vaccine platforms, with the overall ordering RP-3 ≈ CV-3 > CV-2 ≈ RA-1 > C-0. This pattern suggests that immunization regimen is closely associated with the magnitude of humoral responses and is consistent with earlier evidence that booster immunization can enhance humoral protection (21–24). Although the overall pattern remained largely unchanged after additional adjustment for days since vaccination in sensitivity analyses, a potential effect of vaccination recency cannot be excluded. After natural infection, antibody levels increased across all groups and showed a tendency toward convergence. This pattern likely reflects the strong boosting effect of infection itself, which may reduce pre-existing differences between groups; in vaccinated participants, pre-existing immune memory may additionally shape the magnitude of the response (25). These findings extend previous reports largely based on mRNA-vaccinated cohorts by demonstrating that similar boosting and partial convergence also occur in populations predominantly immunized with inactivated, recombinant protein, and adenoviral vector vaccines (10, 26). However, differences in antibody recognition at the variant level persisted. We observed reduced RBD-binding antibody reactivity to BA.5 relative to WT, consistent with prior reports describing Omicron BA.4/BA.5 as being less well recognized by pre-existing serum antibodies (27). Importantly, although absolute antibody levels increased after BTI, the persistently negative BA.5 escape index suggests that cross-protection may arise from broader antibody repertoires and affinity maturation rather than proportional increases in BA.5-specific antibodies alone (28).

### ii. Antibody response, neutralizing activity, and associated factors

We assessed neutralizing activity in a serum subset with higher anti-RBD IgG levels and observed a significant positive correlation between anti-RBD IgG levels and IC50. This finding is consistent with our previous results and with prior reports showing significant associations between anti-S1/RBD IgG levels and neutralization readouts, including IC50 (19, 29). These results suggest concordance between binding antibody levels and functional neutralizing capacity, providing functional support for the humoral findings. Notably, this concordance was stronger in recombinant vaccine recipients than in inactivated vaccine recipients, suggesting platform-specific differences in the relationship between binding and neutralizing responses (30, 31).

We also found that age was associated with WT-specific anti-RBD IgG at T1, but not at T2. This suggests that the factors affecting humoral responses after vaccination and after breakthrough infection may not be the same. However, this finding should be interpreted in a broader clinical context. Besides possible immune changes after infection, the weaker age association at T2 may also reflect differences in infection severity, since more severe COVID-19 has been associated with higher convalescent anti-RBD antibody levels (32–34).

### iii. Analyte-specific changes in the cytokine profile

Compared with humoral measures, cytokines may reflect broader inflammatory and immunoregulatory states, and their interpretation is often analyte-specific and context-dependent (35). In our cohort, cytokine changes from T1 to T2 were not uniform. The Th2-associated cytokine IL-13 decreased consistently within all groups, representing the most stable longitudinal signal, IL-5, IL-6, and IL-10 also declined in several groups, whereas changes in most other cytokines were more limited and were observed only in selected groups. Prior studies in acute moderate-to-severe COVID-19 have reported elevated IL-13 levels associated with adverse outcomes and have suggested that IL-13 may contribute to more severe disease phenotypes (36). However, T2 sampling in this study occurred months after infection, and the widespread decline in IL-13 is more likely to reflect resolution of acute inflammatory signals and waning of acute inflammatory signals during convalescence. Together with the decline in IL-5, IL-6, and IL-10 in some groups, these findings are consistent with attenuation of Th2-associated immune signals during the convalescent phase. In parallel, PCA and heatmap analyses suggested only subtle shifts in the serum cytokine profile after infection, with substantial overlap across vaccine groups and time points. These findings should be interpreted descriptively and do not provide strong evidence for vaccine-group-specific changes in the regulatory or inflammatory milieu beyond the more consistent Th2-/inflammation-related patterns (37–39). Future longitudinal analyses integrating cytokine dynamics with functional measurements of memory T and B cells will be needed to clarify the biological significance of these observations.

### iv. Immune changes after breakthrough infection

Overall, BTI may further enhance humoral immune responses on top of pre-existing vaccine-induced immunity, and may be accompanied by a reduction in some between-platform differences, as well as changes in inflammatory and immunoregulatory markers (40, 41). This pattern differs from the persistent inflammatory signatures reported in some unvaccinated or acutely infected cohorts (42, 43), which more often reflect immune imbalance during the early or prolonged phases of disease. In contrast, our findings suggest that in vaccinated populations, the immune changes observed after BTI are more likely to reflect recovery-related immune contraction or partial normalization after infection, rather than sustained systemic inflammation. This overall pattern of “boosting with partial convergence” is consistent with previous observations following repeated antigen exposure (44–46), but it does not imply complete homogenization of immune responses. Instead, post-infection immunity remained continuously distributed across individuals, with a clear low-tail phenotype.

### v. Identification and implications of the lower-quartile subgroup

We used a quantile-based approach to describe individuals in the lower-quartile of the composite immune index (T2-LQ) to further characterize post-infection immune heterogeneity. In this study, the T2-LQ phenotype remained consistent across alternative standardization strategies and feature sets, and it was unlikely to be driven primarily by differences in follow-up timing. Among the measured covariates, vaccine group and baseline demographic factors did not reliably predict T2-LQ membership. This is broadly consistent with prior large cohort studies: although these factors are associated with anti-S1/RBD antibody levels, they typically explain only a portion of the overall variability, leaving substantial inter-individual differences that are not captured by routine clinical variables (47, 48). Importantly, within the BTI subset, pre-infection WT anti-RBD IgG levels were comparable between T2-LQ and non-LQ individuals, indicating that low-tail status after infection is not simply determined by a low baseline antibody level. It may reflect the combined influence of multiple host- and infection-related factors, including differences in recall response, disease severity, and time since infection (25, 49). From a public health perspective, these findings support continued immune monitoring and may help identify individuals who warrant closer follow-up after infection or vaccination.

### vi. Strengths, limitations, and perspectives

This study’s strengths include its paired-sample design, real-world population base, and comprehensive immune profiling integrating humoral and cytokine parameters. However, limitations include modest sample size, sampling intervals differed inherently across vaccine types, and residual confounding that cannot be fully excluded because vaccine platform was observational rather than randomized. Although we adjusted for sampling intervals and the infection wave was relatively time-concentrated (which reduced variability in waning time), residual confounding due to inter-individual differences in antibody kinetics cannot be fully excluded; IC50 measurements were performed only in a high-anti-RBD IgG subset, which limits the generalizability of neutralization findings to the full cohort; RBD-IgG levels were quantified using an in-house standard, which may limit comparability across studies; the lack of severe case follow-up or functional T−cell assays. Future studies should extend longitudinal observation, include cellular assays, and evaluate clinical protection endpoints to validate predictive biomarkers of durable immunity.

## Conclusions and implications

Our findings provide real-world evidence that vaccine-induced and infection-induced immunity together shape a dynamic, heterogeneous immune landscape. Breakthrough infection may further augment immune responses and partially narrow differences across vaccine platforms, while also being associated with modest shifts in overall immune profiles rather than uniform amplification.

The presence of a post-infection lower-quartile subgroup highlights the potential need for targeted monitoring and adaptive vaccination strategies. These results may inform the development of simplified immune monitoring frameworks and optimized booster regimens tailored to hybrid immunity in the post-pandemic era.

## Data Availability

The original contributions presented in the study are included in the article/**Supplementary Material**. Further inquiries can be directed to the corresponding authors.
