# Supplementary material for: Real-world immune dynamics following COVID-19 vaccination and breakthrough infection: a paired-sample study in Zhejiang Province, China
Source: Front Immunol. 2026 Mar 31;17:1801564. doi: 10.3389/fimmu.2026.1801564 (PMC13077751; doi:10.3389/fimmu.2026.1801564)
Supplement: Supplementary file 1 [file DataSheet1.zip › Supplementary materials/Supplementary files.docx]

Supplementary Material

# Supplementary Methods

## Data preprocessing

Antibody (anti–RBD IgG), neutralization activity (IC50), and cytokine measurements were all modeled on the log10 scale prior to statistical analyses. Sex was included as a categorical covariate and age as a continuous covariate. Unless otherwise specified, all tests were two-sided with a significance threshold of p < 0.05.

## Handling zeros/detection limits and pseudocount sensitivity analyses

For endpoints that included zero values (e.g., IC50 and some cytokines), we applied a log10(X + c) transformation to enable linear-model analyses. Robustness to the choice of pseudocount was assessed by repeating key comparisons across alternative c values; the main conclusions were consistent over a reasonable range of c. (Fig. S1).

## Longitudinal and cross-sectional inference: linear mixed-effects models (LMMs) with Tukey adjustment

For outcomes with repeated measurements from the same participant across time points (e.g., paired analyses at T1 and T2), we used linear mixed-effects models to account for within-subject correlation by including a random intercept for Subject_ID:

Y ~ Vaccine_Group × Timepoint + Age + Sex + (1 | Subject_ID), where Y denotes the log10-transformed endpoint (anti–RBD IgG, IC50, or cytokines). After model fitting, inference was based on estimated marginal means (EMMs). Pairwise comparisons among vaccine groups were performed within each time point, with multiple-comparisons adjustment using Tukey’s method. For cross-sectional comparisons at a single time point (e.g., T1-only or T2-only analyses), we applied the same fixed-effects structure to the corresponding time-specific subset and conducted pairwise group contrasts using the EMM framework with Tukey adjustment.

Model assumptions were evaluated for all adjusted linear and linear mixed-effects models. For linear models, we examined residual distribution, heteroscedasticity, influential observations, and multicollinearity; for mixed-effects models, convergence and singularity were additionally assessed. Diagnostic summaries for all fitted models are presented in Supplementary Table S12.

## Time-adjusted sensitivity models

## Because differences in vaccination schedules and sampling windows may introduce time-dependent confounding due to antibody waning, we fitted time-adjusted sensitivity models to evaluate robustness. The interval from the most recent vaccine dose to T1 sampling (Days_Since_Vax) is now summarized by vaccine group in Supplementary Table S3. For T1 cross-sectional analyses, we additionally included time since the most recent vaccination (Days_Since_Vax, days) as a covariate. For T2 analyses, because samples were collected across multiple clinical sites and dates of infection/positivity were not consistently available, we included the T1–T2 sampling interval (Days_T1_T2, days) as a timing covariate. The direction of between-group effects in these time-adjusted models was consistent with the primary analyses, supporting robustness to time-related confounding (summary results are provided in the Supplementary Table S5).

## FDR adjustment for within-group multi-cytokine comparisons

For cytokine analyses, different multiplicity-adjustment strategies were applied according to the inferential target. For paired longitudinal analyses, linear mixed-effects models were fitted separately for each cytokine using subjects with paired T1 and T2 measurements, and model-derived within-group longitudinal contrasts (T2−T1) were estimated for each vaccine group. Raw *P* values from all estimable cytokine-by-group within-group longitudinal contrasts were pooled and adjusted using the Benjamini–Hochberg procedure to control the false discovery rate. For between-group comparisons of a given cytokine within each time point, pairwise contrasts were adjusted using Tukey’s method. Separately, for within-group cytokine-profile analyses, linear mixed-effects models were fitted within each vaccine group and time point to compare the relative levels of the eight cytokines; pairwise comparisons among cytokines were then performed within each vaccine group × time-point subset, and the resulting *P* values were adjusted using the Benjamini–Hochberg procedure within that subset. Adjusted p values from Benjamini–Hochberg correction are reported as *q* values.

## Software and implementation

Analyses were conducted in R (v4.4.1) running on Windows 11 x64 (build 26100). Key R packages included readxl, dplyr/tidyverse, lme4, lmerTest, emmeans, ggplot2, broom, broom.mixed, performance, car, lmtest, patchwork, and readr. All analysis code, including scripts for model fitting, diagnostics, and figure generation, has been made publicly available and is provided with the Supplementary Materials.

# Supplementary Figures and Tables

## Supplementary Figures


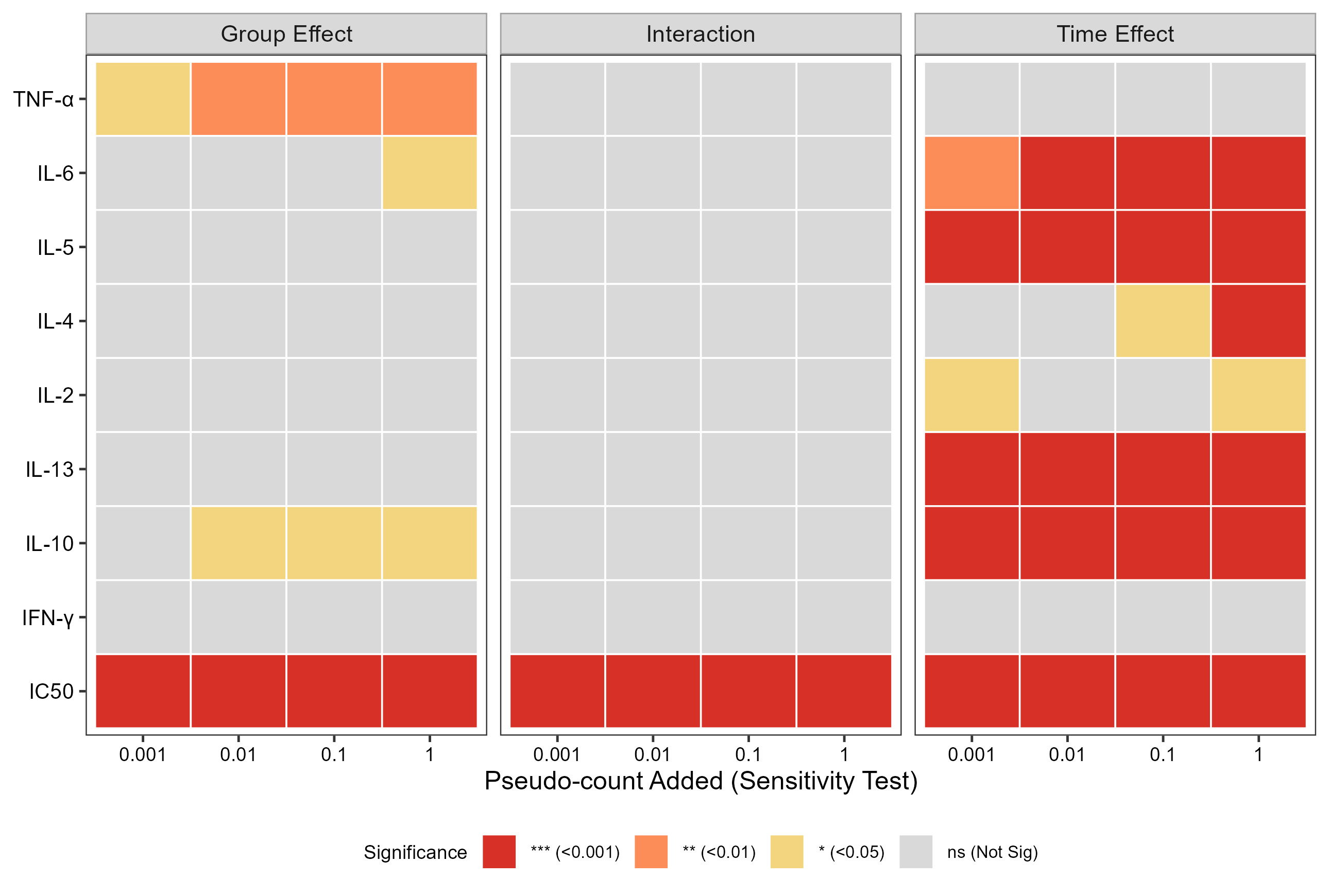


**Supplementary Figure 1. Sensitivity analyses for pseudo-count handling of zero/limit values prior to log_10_ transformation.**

For endpoints containing zero values (including IC50 and cytokines), we applied a log_10_(x+c) transformation and repeated the same linear models (ANCOVA) across pseudo-counts c ∈ {0.001, 0.01, 0.1, 1} to test group effects, time effects, and group-by-time interactions. Colors denote the significance level for the corresponding tests; consistent colors for a given endpoint across c values indicate robustness to the choice of zero-handling strategy.


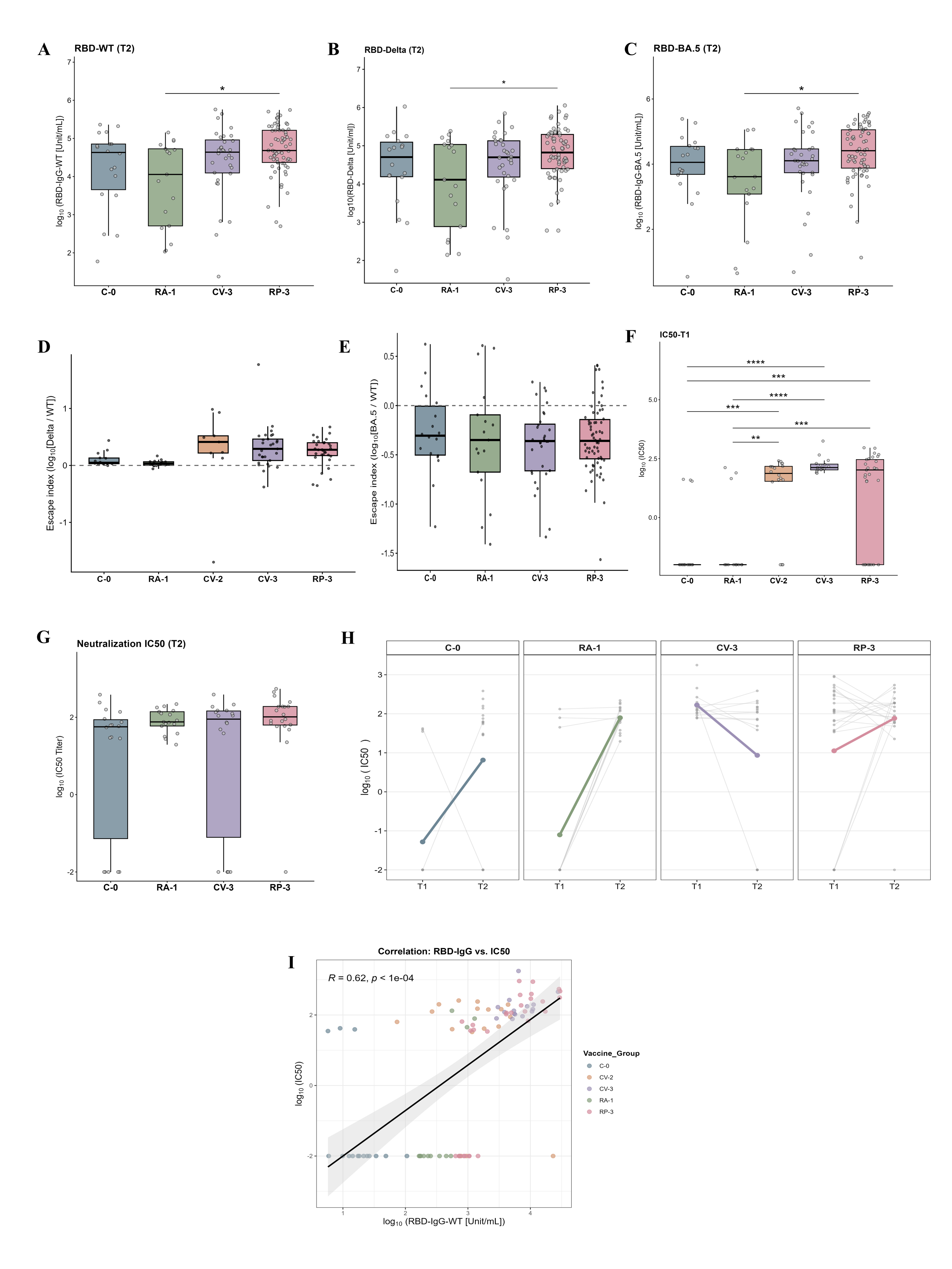


**Supplementary Figure 2. Additional humoral response analyses.**

(A–C) Group comparisons of anti–RBD IgG levels against WT (A), Delta (B) and BA.5 (C) at T2. WT-, Delta-, and BA.5-RBD IgG were measured in all paired BTI participants (N = 133). (D) Delta escape index at T1, defined as the within-individual difference in log_10_-transformed anti–RBD IgG levels between Delta and WT (log_10_[Delta] − log_10_[WT]). (E) BA.5 escape index at T2, defined as log_10_[BA.5] − log_10_[WT]. At T2, the BA.5 escape index was calculated in all paired BTI participants and remained mostly below zero, indicating persistent reduction in BA.5 recognition relative to WT despite overall post-BTI boosting of antibody levels. (F–G) Neutralization activity (IC50) at T1 (F) and T2 (G). (H) Paired longitudinal trajectories of IC50 from T1 to T2. (I) Pearson correlation between log_10_-transformed WT anti–RBD IgG levels and log_10_-transformed IC50. Statistics: if not displayed in the panels, corresponding inference results are provided in Supplementary Tables S4–S5. Asterisks indicate pairwise between-group comparisons at T1/T2 based on estimated marginal means from a linear mixed-effects model, with Tukey adjustment for multiple comparisons (**p* < 0.05, ***p* < 0.01, ****p* < 0.001, *****p* < 0.0001). Sample sizes for each group and panel are provided in Tables S4–S5. (RA-1, one dose of adenoviral vector vaccine; CV-2, two doses of inactivated vaccine; CV-3, three doses of inactivated vaccine; RP-3, three doses of recombinant protein vaccine and C-0, unvaccinated group).


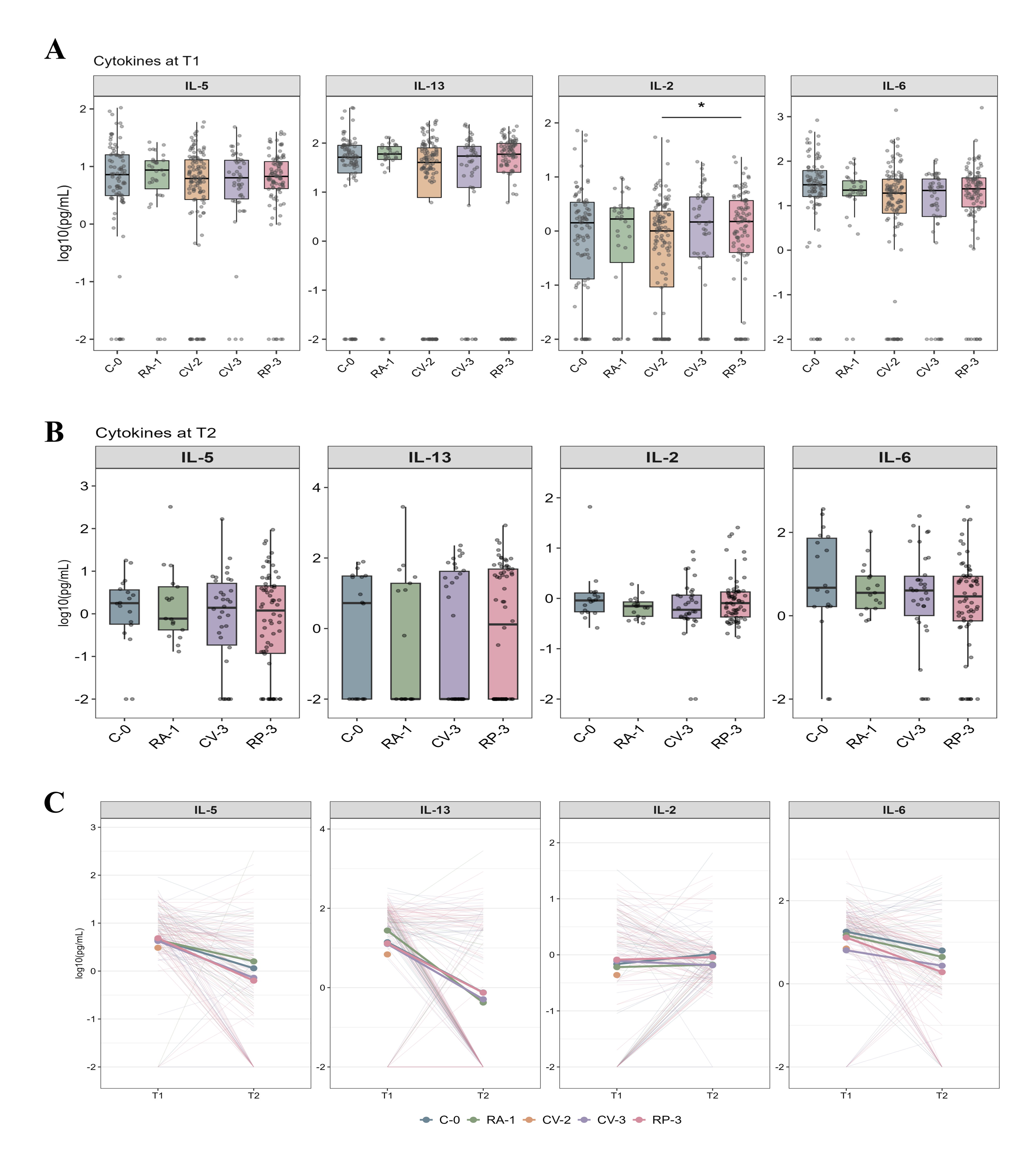


**Supplementary Figure 3. Serum cytokine supplementation analysis**

(A) Distributions of IL-2, IL-5, IL-6, and IL-13 levels across vaccine groups at T1.

(B) Distributions of IL-2, IL-5, IL-6, and IL-13 levels across vaccine groups at T2.

(C) Within-participant paired trajectories of IL-2, IL-5, IL-6, and IL-13 from T1 to T2, showing individual-level changes and group mean trends; no significance annotations are overlaid in this panel.

Cross-sectional between-group inference was based on linear mixed-effects models with Tukey-adjusted pairwise comparisons within each time point (**p* < 0.05, ***p* < 0.01, ****p* < 0.001, *****p* < 0.0001); inferential pairwise comparisons for within-group cytokine profiles were adjusted using the Benjamini–Hochberg FDR procedure and sample sizes for each panel are provided in Table S7. (RA-1, one dose of adenoviral vector vaccine; CV-2, two doses of inactivated vaccine; CV-3, three doses of inactivated vaccine; RP-3, three doses of recombinant protein vaccine and C-0, unvaccinated group).


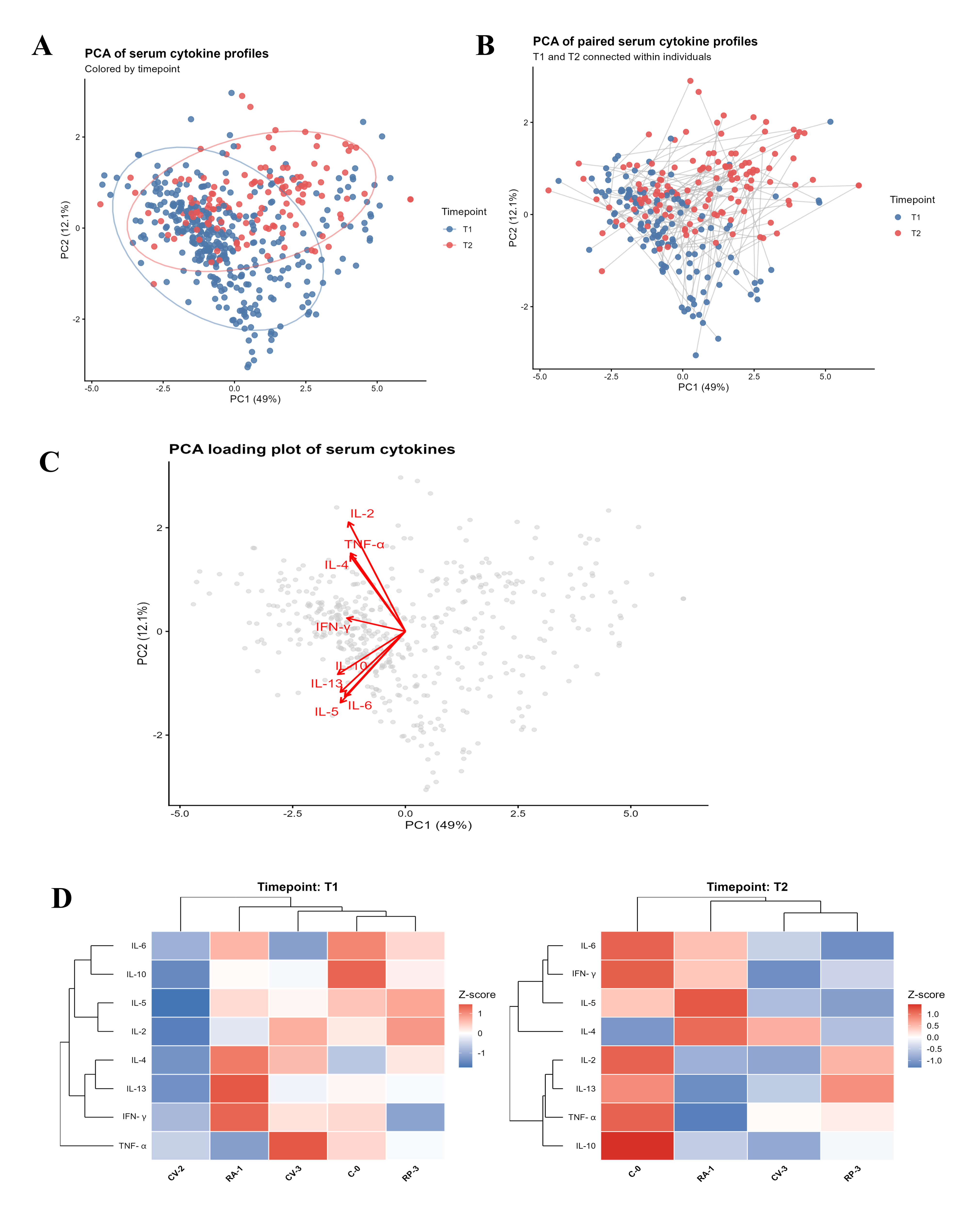


**Supplementary Figure 4. Exploratory PCA and heatmap visualization of serum cytokine profiles at T1 and T2.**

(A) PCA of all available serum cytokine samples, colored by timepoint (T1 vs T2).

(B) PCA of participants with paired serum samples collected at both T1 and T2; measurements from the same individual are connected by grey lines.

(C) PCA loading plot showing the contribution of each cytokine to the first two principal components. Cytokine values were log10(x + 0.01)-transformed before analysis and were centered and scaled for PCA. PC1 and PC2 explained 49.0% and 12.1% of the total variance, respectively. The analysis showed substantial overlap across time points, consistent with modest reshaping rather than strong global reorganization of the serum cytokine landscape after BTI.

(D) Heatmap showing the relative levels of eight cytokines across vaccine groups at T1 and T2 based on Z-score-normalized log10-transformed values.

Cytokine values were log10(x + 0.01)-transformed before analysis. For PCA, values were further centered and scaled. PC1 and PC2 explained 49.0% and 12.1% of the total variance, respectively. Overall, PCA showed substantial overlap between T1 and T2, with only a modest shift after BTI rather than strong global separation. The heatmap provides a complementary descriptive view of relative cytokine patterns across groups and time points.


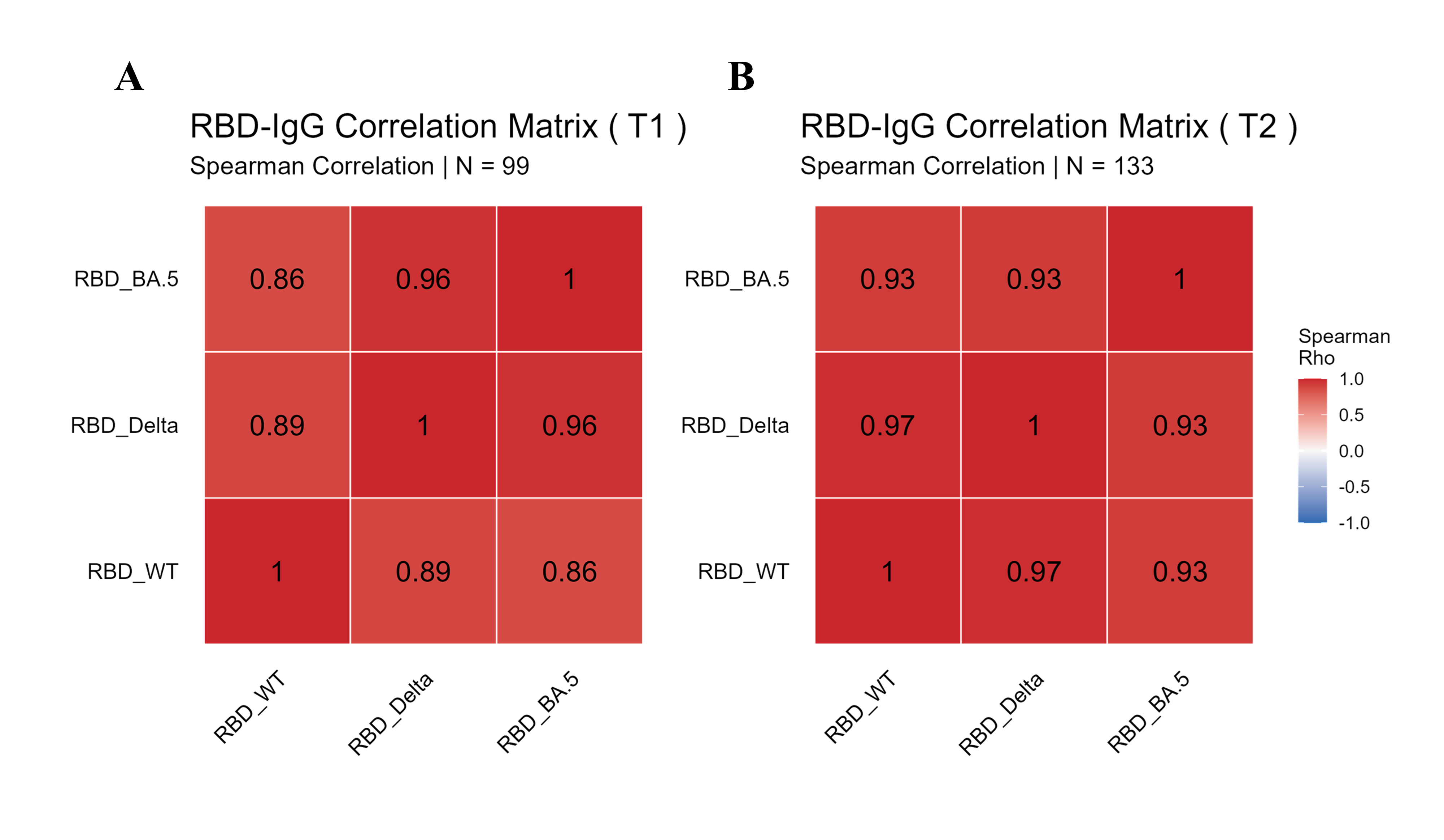


**Supplementary Figure 5. Internal consistency of variant-specific RBD-IgG measurements at T1 and T2.**

Spearman correlation matrices (ρ) of log_10_-transformed WT-, Delta-, and BA.5-specific RBD-IgG levels are shown separately for T1(A) and T2(B). Color intensity indicates the magnitude of pairwise correlations; two-sided P values for Spearman correlations are provided in the corresponding table.

**
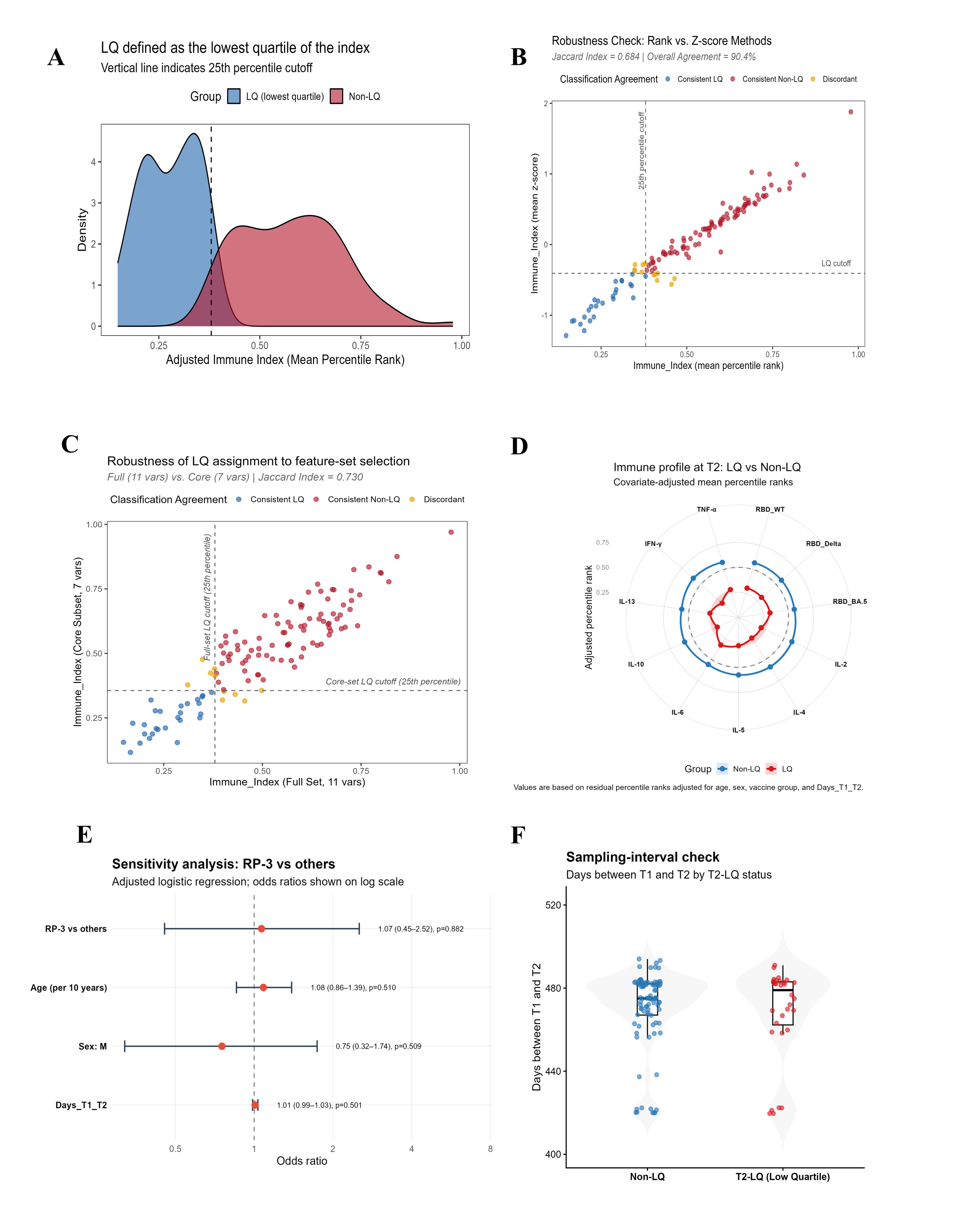
**

**Supplementary Figure 6. Construction and robustness of the covariate-adjusted T2 immune index and lower-quartile subgroup (T2-LQ).**

(A) Density distribution of the covariate-adjusted composite immune index at T2 (Immune Index), constructed as the mean percentile rank of model-adjusted residuals across 11 metrics. The dashed line marks the 25th percentile cutoff used to define T2-LQ.

(B) Robustness to standardization strategy: comparison of the index based on residual percentile ranks (x axis) versus the index based on residual Z-scores (y axis). Dashed lines indicate the LQ cutoffs for each index. Point colors denote concordance of LQ classification between methods: concordant LQ (blue), concordant non-LQ (red), and discordant (orange). Overall agreement was 90.4% (Jaccard index = 0.684).

(C) Robustness to the feature set: comparison of the full 11-metric index (x axis) versus a prespecified 7-metric core index (y axis; three RBD-IgG measures plus IFN-γ, TNF-α, IL-4, and IL-10). Both indices were constructed using the same pipeline. Dashed lines indicate the respective LQ cutoffs, and point colors are as in (B). LQ membership overlapped substantially between indices (Jaccard = 0.730).

(D) The radar plot summarizes relative levels at T2 for the T2-LQ and non-LQ groups across humoral measures (WT/Delta/BA.5 RBD-IgG and IC50) and eight cytokines. Values on each axis represent covariate-adjusted group mean “relative scores” (residual Z-scores after adjusting for age, sex, and vaccine group; 0 denotes the overall mean), with lower values indicating lower relative levels. This plot is intended to summarize overall immune profile differences between groups and is not used for significance inference on individual markers.

(F) Sensitivity analysis: all BTI cases were pooled and compared with the infection-only (unvaccinated infected) group as the reference in a multivariable logistic regression model for T2-LQ membership. Models adjusted for age (per 10 years), sex, and the T1–T2 sampling interval; odds ratios (ORs) with 95% confidence intervals are shown on a log scale.

(F) Follow-up interval check: distribution of the T1–T2 sampling interval in T2-LQ versus non-LQ participants. Violin/box plots show the interval (days) to assess whether stratification could be influenced by systematic differences in follow-up timing. Distributions were similar between groups, and the interval was also adjusted for as a covariate during index construction.

## Supplementary Tables

**Supplementary Table 1. The number of vaccine immunization among 370 participants.**

| **Number of people** | **C-0** | **RA-1** | **CV-2** | **CV-3** | **RP-3** | **Total** |
| --- | --- | --- | --- | --- | --- | --- |
| Hangzhou | 22 | 4 | 10 | 1 | 8 | 45 |
| Ningbo | 14 | 0 | 15 | 9 | 22 | 60 |
| Wenzhou | 11 | 1 | 5 | 2 | 8 | 27 |
| Huzhou | 13 | 0 | 7 | 7 | 9 | 36 |
| Jiaxing | 0 | 0 | 0 | 0 | 3 | 3 |
| Shaoxing | 6 | 2 | 18 | 5 | 4 | 35 |
| Jinhua | 12 | 2 | 18 | 3 | 13 | 48 |
| Quzhou | 1 | 4 | 16 | 8 | 2 | 31 |
| Zhoushan | 0 | 17 | 0 | 0 | 7 | 24 |
| Taizhou | 1 | 0 | 9 | 11 | 16 | 37 |
| Lishui | 5 | 1 | 16 | 1 | 1 | 24 |
| **Total** | 85 | 31 | 114 | 47 | 93 | 370 |

**Supplementary Table 2. Baseline characteristics of participants with paired sera, stratified by vaccine group.**

| **Variable** | **C-0** | **RA-1** | **CV-3** | **RP-3** |
| --- | --- | --- | --- | --- |
| n (paired) | 18 | 17 | 32 | 66 |
| Female, n (%) | 8 (44.44%) | 10 (58.82%) | 14 (43.75%) | 37 (56.06%) |
| Age, median (IQR) | 76 (64,79) | 35 (33,54) | 53 (32,62) | 55.5 (40.25,65.75) |
| Days_Since_Vax | / | 186 (175, 190) | 45 (37.75, 55) | 136 (131.5,145.25) |
| Days_T1_T2 | 462.5 (420.5, 486.5) | 483 (473, 484) | 468 (456, 481) | 479 (472,482) |

**Supplementary Table 3. Days from the most recent vaccine dose to T1 sampling (Days_Since_Vax) by vaccine group.**

| **Vaccine group** | **N** | **Days_Since_Vax, median (IQR)** | ***P* Value** |
| --- | --- | --- | --- |
| RA-1 | 31 | 189 (176–203.5) | / |
| CV-2 | 114 | 159 (105.75–187) | / |
| CV-3 | 47 | 44 (23.5–55) | / |
| RP-3 | 93 | 136 (131–149) | / |
| Overall comparison | / | / | <0.0001 |

**Supplementary Table 4. Serum RBD-IgG positive rate by Age and Vaccination Status.**

| **Group** | | **T1** | | | **T2** | | |
| --- | --- | --- | --- | --- | --- | --- | --- |
| **Immune species** | **Age** | **Survey number** | **Positive number** | **Seroconversion rate** | **Survey number** | **Positive number** | **seroconversion rate** |
| C-0 | 3-19 | 28 | 4 | 14.29 | / | / | / |
|  | 20-39 | 16 | 0 | 0.00 | 2.00 | 2.00 | 100.00 |
|  | 40-59 | 12 | 0 | 0.00 | 1.00 | 1.00 | 100.00 |
|  | ≥60 | 29 | 1 | 3.45 | 15.00 | 14.00 | 93.33 |
|  | **Total** | 85 | 5 | 5.88 | 18.00 | 17.00 | 94.44 |
| RA-1 | 3-19 | 1 | 1 | 100.00 | 1.00 | 1.00 | 100.00 |
|  | 20-39 | 16 | 9 | 56.25 | 10.00 | 10.00 | 100.00 |
|  | 40-59 | 9 | 6 | 66.67 | 3.00 | 3.00 | 100.00 |
|  | ≥60 | 5 | 3 | 60.00 | 3.00 | 3.00 | 100.00 |
|  | **Total** | 31 | 19 | 61.30 | 17.00 | 17.00 | 100.00 |
| CV-2 | 3-19 | 33 | 28 | 84.85 | / | / | / |
|  | 20-39 | 16 | 11 | 68.75 | / | / | / |
|  | 40-59 | 19 | 9 | 47.37 | / | / | / |
|  | ≥60 | 46 | 27 | 58.70 | / | / | / |
|  | **Total** | 114 | 75 | 65.79 | / | / | / |
| CV-3 | 3-19 | 8 | 8 | 100.00 | 4.00 | 3.00 | 75.00 |
|  | 20-39 | 11 | 11 | 100.00 | 8.00 | 8.00 | 100.00 |
|  | 40-59 | 10 | 8 | 80.00 | 9.00 | 9.00 | 100.00 |
|  | ≥60 | 18 | 17 | 94.44 | 11.00 | 11.00 | 100.00 |
|  | **Total** | 47 | 44 | 93.62 | 32.00 | 31.00 | 96.88 |
| RP-3 | 3-19 | 2 | 2 | 100.00 | 1.00 | 1.00 | 100.00 |
|  | 20-39 | 18 | 16 | 88.89 | 15.00 | 15.00 | 100.00 |
|  | 40-59 | 34 | 31 | 91.18 | 22.00 | 22.00 | 100.00 |
|  | ≥60 | 39 | 35 | 89.74 | 28.00 | 28.00 | 100.00 |
|  | **Total** | 93 | 84 | 90.32 | 66.00 | 66.00 | 100.00 |

**Supplementary Table 5. Time-adjusted sensitivity analyses for anti–RBD IgG responses at T1 and T2.**

| Outcome | Variable | Estimate (95% CI) / N | *P* Value |
| --- | --- | --- | --- |
| **Panel: Panel A: T1 Adjusted Model (Ref=RA-1)** | | | |
| RBD_BA.5 | CV-2 vs RA-1 | 1.24 (0.86, 1.63) | < 0.001 |
| RBD_BA.5 | CV-3 vs RA-1 | 1.53 (0.97, 2.09) | < 0.001 |
| RBD_BA.5 | RP-3 vs RA-1 | 1.73 (1.42, 2.03) | < 0.001 |
| RBD_BA.5 | Days_Since_Vax | -0.00 (-0.00, 0.00) | 0.92 |
| RBD_Delta | CV-2 vs RA-1 | 1.40 (1.08, 1.72) | < 0.001 |
| RBD_Delta | CV-3 vs RA-1 | 1.71 (1.24, 2.18) | < 0.001 |
| RBD_Delta | RP-3 vs RA-1 | 1.78 (1.53, 2.03) | < 0.001 |
| RBD_Delta | Days_Since_Vax | 0.00 (-0.00, 0.00) | 0.75 |
| RBD_WT | CV-2 vs RA-1 | 0.11 (-0.16, 0.38) | 0.43 |
| RBD_WT | CV-3 vs RA-1 | 0.83 (0.42, 1.24) | < 0.001 |
| RBD_WT | RP-3 vs RA-1 | 0.79 (0.50, 1.08) | < 0.001 |
| RBD_WT | Days_Since_Vax | -0.00 (-0.00, -0.00) | 0.05 |
| **Panel: Panel B: T2 Adjusted Model (Ref=C-0)** | | | |
| RBD_BA.5 | CV-3 vs C-0 | 0.12 (-0.56, 0.80) | 0.72 |
| RBD_BA.5 | RA-1 vs C-0 | -0.30 (-1.11, 0.51) | 0.46 |
| RBD_BA.5 | RP-3 vs C-0 | 0.52 (-0.12, 1.16) | 0.11 |
| RBD_BA.5 | Days_T1_T2 | 0.00 (-0.01, 0.01) | 0.95 |
| RBD_Delta | CV-3 vs C-0 | 0.09 (-0.50, 0.68) | 0.76 |
| RBD_Delta | RA-1 vs C-0 | -0.28 (-0.98, 0.42) | 0.43 |
| RBD_Delta | RP-3 vs C-0 | 0.47 (-0.08, 1.02) | 0.10 |
| RBD_Delta | Days_T1_T2 | -0.00 (-0.01, 0.01) | 0.67 |
| RBD_WT | CV-3 vs C-0 | 0.21 (-0.37, 0.79) | 0.47 |
| RBD_WT | RA-1 vs C-0 | -0.30 (-0.99, 0.39) | 0.39 |
| RBD_WT | RP-3 vs C-0 | 0.57 (0.03, 1.11) | 0.04 |
| RBD_WT | Days_T1_T2 | -0.00 (-0.01, 0.01) | 0.91 |
| **Panel: Panel C: Data Coverage** | | | |
| RBD_BA.5 | Sample Size | N=85(T1) | / |
| RBD_BA.5 | Sample Size | N=125 (T2) | / |
| RBD_Delta | Sample Size | N=85 (T1) | / |
| RBD_Delta | Sample Size | N=125 (T2) | / |
| RBD_WT | Sample Size | N=285 (T1) | / |
| RBD_WT | Sample Size | N=125 (T2) | / |
| Note: Table S4 demonstrates robustness against time-related confounding. | | | |

**Supplementary Table 6. Longitudinal mixed-effects model inference for anti–RBD IgG responses from T1 to T2 (paired samples).**

| **Outcome** | **Group / Contrast** | **Metric** | **Estimate (95% CI) / N** | ***P* Value** |
| --- | --- | --- | --- | --- |
| **Panel A: Longitudinal Change [Δ** β=**Δlog_10_ (T2−T1)]** | | | | |
| RBD_WT | C-0 | **Δ** β | 2.90 (2.39, 3.41) | < 0.001 |
| RBD_WT | RA-1 | **Δ** β | 1.59 (1.07, 2.11) | < 0.001 |
| RBD_WT | CV-3 | **Δ** β | 1.23 (0.84, 1.61) | < 0.001 |
| RBD_WT | RP-3 | **Δ** β | 1.83 (1.56, 2.09) | < 0.001 |
| RBD_Delta | C-0 | **Δ** β | 2.88 (2.40, 3.36) | < 0.001 |
| RBD_Delta | RA-1 | **Δ** β | 1.72 (1.24, 2.20) | < 0.001 |
| RBD_Delta | CV-3 | **Δ** β | 0.84 (0.44, 1.24) | < 0.001 |
| RBD_Delta | RP-3 | **Δ** β | 0.83 (0.44, 1.22) | < 0.001 |
| RBD_BA5 | C-0 | **Δ** β | 3.04 (2.49, 3.58) | < 0.001 |
| RBD_BA5 | RA-1 | **Δ** β | 1.72 (1.17, 2.26) | < 0.001 |
| RBD_BA5 | CV-3 | **Δ** β | 0.89 (0.43, 1.34) | < 0.001 |
| RBD_BA5 | RP-3 | **Δ** β | 0.96 (0.52, 1.41) | < 0.001 |
| **Panel B: Between-group Comparisons (Tukey)** | | | | |
| RBD_WT | T1: (C-0) - (RA-1) | Difference | -0.80 (-1.51, -0.09) | 0.021 |
| RBD_WT | T1: (C-0) - (CV-3) | Difference | -1.77 (-2.39, -1.15) | < 0.001 |
| RBD_WT | T1: (C-0) - (RP-3) | Difference | -1.51 (-2.06, -0.96) | < 0.001 |
| RBD_WT | T1: (RA-1) - (CV-3) | Difference | -0.97 (-1.58, -0.36) | < 0.001 |
| RBD_WT | T1: (RA-1) - (RP-3) | Difference | -0.71 (-1.26, -0.15) | 0.006 |
| RBD_WT | T1: (CV-3) - (RP-3) | Difference | 0.26 (-0.18, 0.70) | 0.41 |
| RBD_WT | T2: (C-0) - (RA-1) | Difference | 0.51 (-0.20, 1.22) | 0.256 |
| RBD_WT | T2: (C-0) - (CV-3) | Difference | -0.10 (-0.72, 0.52) | 0.976 |
| RBD_WT | T2: (C-0) - (RP-3) | Difference | -0.44 (-0.99, 0.11) | 0.171 |
| RBD_WT | T2: (RA-1) - (CV-3) | Difference | -0.61 (-1.21, 0.00) | 0.05 |
| RBD_WT | T2: (RA-1) - (RP-3) | Difference | -0.95 (-1.50, -0.39) | < 0.001 |
| RBD_WT | T2: (CV-3) - (RP-3) | Difference | -0.34 (-0.78, 0.10) | 0.192 |
| RBD_Delta | T1: (C-0) - (RA-1) | Difference | -0.63 (-1.33, 0.07) | 0.094 |
| RBD_Delta | T1: (C-0) - (CV-3) | Difference | -2.27 (-2.89, -1.65) | < 0.001 |
| RBD_Delta | T1: (C-0) - (RP-3) | Difference | -2.32 (-2.97, -1.68) | < 0.001 |
| RBD_Delta | T1: (RA-1) - (CV-3) | Difference | -1.64 (-2.23, -1.05) | < 0.001 |
| RBD_Delta | T1: (RA-1) - (RP-3) | Difference | -1.70 (-2.28, -1.11) | < 0.001 |
| RBD_Delta | T1: (CV-3) - (RP-3) | Difference | -0.05 (-0.59, 0.48) | 0.994 |
| RBD_Delta | T2: (C-0) - (RA-1) | Difference | 0.53 (-0.17, 1.23) | 0.200 |
| RBD_Delta | T2: (C-0) - (CV-3) | Difference | -0.23 (-0.86, 0.39) | 0.762 |
| RBD_Delta | T2: (C-0) - (RP-3) | Difference | -0.28 (-0.92, 0.36) | 0.668 |
| RBD_Delta | T2: (RA-1) - (CV-3) | Difference | -0.77 (-1.36, -0.17) | 0.005 |
| RBD_Delta | T2: (RA-1) - (RP-3) | Difference | -0.81 (-1.40, -0.23) | 0.002 |
| RBD_Delta | T2: (CV-3) - (RP-3) | Difference | -0.05 (-0.58, 0.49) | 0.996 |
| RBD_BA5 | T1: (C-0) - (RA-1) | Difference | -0.76 (-1.55, 0.04) | 0.067 |
| RBD_BA5 | T1: (C-0) - (CV-3) | Difference | -2.34 (-3.05, -1.63) | < 0.001 |
| RBD_BA5 | T1: (C-0) - (RP-3) | Difference | -2.43 (-3.16, -1.69) | < 0.001 |
| RBD_BA5 | T1: (RA-1) - (CV-3) | Difference | -1.58 (-2.25, -0.91) | < 0.001 |
| RBD_BA5 | T1: (RA-1) - (RP-3) | Difference | -1.67 (-2.34, -1.00) | < 0.001 |
| RBD_BA5 | T1: (CV-3) - (RP-3) | Difference | -0.09 (-0.70, 0.52) | 0.981 |
| RBD_BA5 | T2: (C-0) - (RA-1) | Difference | 0.56 (-0.24, 1.35) | 0.264 |
| RBD_BA5 | T2: (C-0) - (CV-3) | Difference | -0.19 (-0.90, 0.52) | 0.900 |
| RBD_BA5 | T2: (C-0) - (RP-3) | Difference | -0.35 (-1.09, 0.38) | 0.591 |
| RBD_BA5 | T2: (RA-1) - (CV-3) | Difference | -0.75 (-1.42, -0.07) | 0.023 |
| RBD_BA5 | T2: (RA-1) - (RP-3) | Difference | -0.91 (-1.58, -0.24) | 0.003 |
| RBD_BA5 | T2: (CV-3) - (RP-3) | Difference | -0.17 (-0.78, 0.45) | 0.896 |
| **Panel C: Paired Sample Size** | | | | |
| RBD_WT | C-0 | Sample Size | N=18 (Paired) | / |
| RBD_WT | RA-1 | Sample Size | N=17 (Paired) | / |
| RBD_WT | CV-3 | Sample Size | N=32 (Paired) | / |
| RBD_WT | RP-3 | Sample Size | N=66 (Paired) | / |
| RBD_Delta | C-0 | Sample Size | N=14 (Paired) | / |
| RBD_Delta | RA-1 | Sample Size | N=14 (Paired) | / |
| RBD_Delta | CV-3 | Sample Size | N=20 (Paired) | / |
| RBD_Delta | RP-3 | Sample Size | N=21 (Paired) | / |
| RBD_BA5 | C-0 | Sample Size | N=14 (Paired) | / |
| RBD_BA5 | RA-1 | Sample Size | N=14 (Paired) | / |
| RBD_BA5 | CV-3 | Sample Size | N=20 (Paired) | / |
| RBD_BA5 | RP-3 | Sample Size | N=21 (Paired) | / |
| Note: Table S6 presents longitudinal inferential statistics. CIs for Delta and Tukey are calculated from SE. | | | | |

**Supplementary Table 7. Primary and vaccination-timing sensitivity analyses of cytokine levels at T1 and T2.**

| **Model term** | **IFNγ** | | **TNFα** | **IL4** | **IL10** | **IL13** | **IL2** | **IL5** | **IL6** |
| --- | --- | --- | --- | --- | --- | --- | --- | --- | --- |
| **T1-Primary model (all groups)** | | | | | | | | | |
| Vaccine_Group, *P* | 0.665 | 0.018 | | 0.048 | 0.052 | 0.292 | 0.082 | 0.550 | 0.080 |
| Age, *P* | 0.470 | 0.002 | | <0.001 | 0.171 | 0.199 | <0.001 | 0.778 | 0.314 |
| Sex, *P* | 0.829 | 0.780 | | 0.468 | 0.045 | 0.410 | 0.677 | 0.201 | 0.999 |
| **T1-Sensitivity model (vaccinated only)** | | | | | | | | | |
| Vaccine_Group, *P* | 0.287 | 0.386 | | 0.032 | 0.274 | 0.164 | 0.100 | 0.336 | 0.155 |
| Age, *P* | 0.936 | 0.262 | | <0.001 | 0.524 | 0.169 | 0.002 | 0.955 | 0.554 |
| Sex, *P* | 0.273 | 0.964 | | 0.7355 | 0.038 | 0.361 | 0.264 | 0.388 | 0.995 |
| Days_Since_Vax *P* | 0.051 | 0.387 | | 0.694 | 0.570 | 0.384 | 0.462 | 0.168 | 0.417 |
| **T2-Primary model (all groups)** | | | | | | | | | |
| Vaccine_Group, *P* | 0.568 | 0.163 | | 0.510 | 0.076 | 0.916 | 0.107 | 0.473 | 0.292 |
| Age, *P* | 0.379 | 0.580 | | 0.052 | 0.156 | 0.673 | 0.043 | 0.212 | 0.853 |
| Sex, *P* | 0.236 | 0.197 | | 0.020 | 0.796 | 0.341 | 0.691 | 0.454 | 0.457 |
| **T2-Sensitivity model (vaccinated only)** | | | | | | | | | |
| Vaccine_Group, *P* | 0.988 | 0.046 | | 0.349 | 0.426 | 0.302 | 0.186 | 0.711 | 0.333 |
| Age, *P* | 0.220 | 0.182 | | 0.044 | 0.043 | 0.467 | 0.036 | 0.112 | 0.309 |
| Sex, *P* | 0.190 | 0.357 | | 0.025 | 0.717 | 0.441 | 0.545 | 0.908 | 0.529 |
| Days_Since_Vax *P* | 0.939 | 0.079 | | 0.658 | 0.361 | 0.128 | 0.781 | 0.898 | 0.243 |

**Supplementary Table 8. Significant pairwise comparisons from EMM-based Tukey tests for cytokine analyses.**

| **Timepoint** | **Model** | **Cytokine** | **Significant comparison** | ***P* Value** |
| --- | --- | --- | --- | --- |
| T1 | Primary | TNF-α | CV-3 vs RA-1 | 0.032 |
| T1 | Primary | TNF-α | CV-3 vs CV-2 | 0.029 |
| T1 | Primary | IL-10 | C-0 vs CV-2 | 0.026 |
| T1 | Primary | IL-2 | RP-3 vs CV-2 | 0.045 |
| T2 | Sensitivity | TNF-α | RP-3 vs RA-1 | 0.038 |

**Supplementary Table 9. Within-group longitudinal cytokine changes from T1 to T2 based on age- and sex-adjusted linear mixed-effects models with BH-FDR correction.**

| **Cytokine** | **Group** | **Δlog_10_ (T2−T1)** | **Back-transformed ratio (10^Δ^)** | ***P* Value** | ***q* value** |
| --- | --- | --- | --- | --- | --- |
| IL-2 | C-0 | 0.612 | 4.09 | 0.012 | 0.033 |
| IL-2 | RA-1 | 0.164 | 1.46 | 0.509 | 0.651 |
| IL-2 | CV-3 | -0.107 | 0.78 | 0.543 | 0.669 |
| IL-2 | RP-3 | 0.013 | 1.03 | 0.921 | 0.921 |
| IFN-γ | C-0 | -0.132 | 0.74 | 0.723 | 0.771 |
| IFN-γ | RA-1 | -0.44 | 0.36 | 0.253 | 0.385 |
| IFN-γ | CV-3 | -0.486 | 0.33 | 0.075 | 0.126 |
| IFN-γ | RP-3 | -0.094 | 0.81 | 0.630 | 0.695 |
| TNF-α | C-0 | 1.262 | 18.27 | 0.011 | 0.031 |
| TNF-α | RA-1 | 0.113 | 1.3 | 0.822 | 0.849 |
| TNF-α | CV-3 | -0.186 | 0.65 | 0.600 | 0.686 |
| TNF-α | RP-3 | 0.295 | 1.97 | 0.248 | 0.385 |
| IL-4 | C-0 | -0.27 | 0.54 | 0.421 | 0.606 |
| IL-4 | RA-1 | -0.237 | 0.58 | 0.493 | 0.651 |
| IL-4 | CV-3 | -0.19 | 0.65 | 0.436 | 0.606 |
| IL-4 | RP-3 | -0.403 | 0.4 | 0.023 | 0.048 |
| IL-5 | C-0 | -0.705 | 0.2 | 0.020 | 0.045 |
| IL-5 | RA-1 | -0.6 | 0.25 | 0.053 | 0.094 |
| IL-5 | CV-3 | -0.895 | 0.13 | < 0.001 | < 0.001 |
| IL-5 | RP-3 | -1.001 | 0.1 | < 0.001 | < 0.001 |
| IL-6 | C-0 | -0.645 | 0.23 | 0.051 | 0.094 |
| IL-6 | RA-1 | -0.768 | 0.17 | 0.024 | 0.048 |
| IL-6 | CV-3 | -0.563 | 0.27 | 0.019 | 0.045 |
| IL-6 | RP-3 | -0.973 | 0.11 | < 0.001 | < 0.001 |
| IL-10 | C-0 | -0.086 | 0.82 | 0.584 | 0.686 |
| IL-10 | RA-1 | -0.535 | 0.29 | 0.002 | 0.005 |
| IL-10 | CV-3 | -0.408 | 0.39 | < 0.001 | 0.002 |
| IL-10 | RP-3 | -0.267 | 0.54 | 0.001 | 0.005 |
| IL-13 | C-0 | -1.545 | 0.03 | 0.003 | 0.008 |
| IL-13 | RA-1 | -1.961 | 0.01 | < 0.001 | 0.001 |
| IL-13 | CV-3 | -1.591 | 0.03 | < 0.001 | < 0.001 |
| IL-13 | RP-3 | -1.136 | 0.07 | < 0.001 | < 0.001 |
| Note: Only participants with paired T1 and T2 measurements for a given cytokine were included. Linear mixed-effects models were fitted with fixed effects for vaccine group, timepoint, age, and sex, and a random intercept for Subject_ID. Effect sizes are model-derived within-group longitudinal contrasts, expressed as Δlog10(T2−T1). Back-transformed ratios were calculated as 10^Δ. For the within-group longitudinal analyses, raw *p* values from all estimable cytokine-by-group T2−T1 contrasts were pooled and adjusted using the Benjamini-Hochberg false discovery rate procedure. The CV-2 group was not included because paired T1 and T2 measurements were unavailable for this subgroup. | | | | | |

**Supplementary Table 10. Linear mixed-effects model-based inference on relative cytokine abundance profiles within each vaccine group at T1 and T2**

| **Timepoint** | | **Vaccine group** | | | **N** | | **N_rows_used** | | |
| --- | --- | --- | --- | --- | --- | --- | --- | --- | --- |
| 1. **Coverage information** | | | | | | | | | |
| T1 | | C-0 | | | 85 | | 680 | | |
| T1 | | CV-2 | | | 114 | | 912 | | |
| T1 | | CV-3 | | | 47 | | 376 | | |
| T1 | | RA-1 | | | 31 | | 248 | | |
| T1 | | RP-3 | | | 93 | | 744 | | |
| T2 | | C-0 | | | 18 | | 144 | | |
| T2 | | CV-3 | | | 34 | | 272 | | |
| T2 | | RA-1 | | | 17 | | 136 | | |
| T2 | | RP-3 | | | 66 | | 528 | | |
| 1. **Model-estimated marginal means (log10) and CLD** | | | | | | | | | |
| **Timepoint** | **Vaccine group** | | **N** | **Cytokine** | | **EMM (log10), 95% CI** | | **CLD** | ***q* value** |
| T1 | C-0 | | 85 | IFN-γ | | 0.74 (0.48, 1.00) | | a | < 0.001 |
| T1 | CV-2 | | 114 | IFN-γ | | 0.59 (0.36, 0.83) | | bc | < 0.001 |
| T1 | CV-3 | | 47 | IFN-γ | | 0.72 (0.40, 1.05) | | ab | < 0.001 |
| T1 | RA-1 | | 31 | IFN-γ | | 0.86 (0.48, 1.25) | | be | < 0.001 |
| T1 | RP-3 | | 93 | IFN-γ | | 0.54 (0.30, 0.78) | | ad | < 0.001 |
| T1 | C-0 | | 85 | IL-10 | | 0.47 (0.21, 0.73) | | a | < 0.001 |
| T1 | CV-2 | | 114 | IL-10 | | 0.22 (-0.02, 0.45) | | a | < 0.001 |
| T1 | CV-3 | | 47 | IL-10 | | 0.34 (0.01, 0.66) | | bc | 0.0015 |
| T1 | RA-1 | | 31 | IL-10 | | 0.35 (-0.03, 0.74) | | a | < 0.001 |
| T1 | RP-3 | | 93 | IL-10 | | 0.35 (0.11, 0.58) | | de | < 0.001 |
| T1 | C-0 | | 85 | IL-13 | | 1.14 (0.88, 1.40) | | b | < 0.001 |
| T1 | CV-2 | | 114 | IL-13 | | 0.84 (0.61, 1.08) | | c | < 0.001 |
| T1 | CV-3 | | 47 | IL-13 | | 1.10 (0.77, 1.42) | | a | < 0.001 |
| T1 | RA-1 | | 31 | IL-13 | | 1.44 (1.05, 1.82) | | c | < 0.001 |
| T1 | RP-3 | | 93 | IL-13 | | 1.10 (0.86, 1.34) | | b | < 0.001 |
| T1 | C-0 | | 85 | IL-2 | | -0.17 (-0.43, 0.09) | | c | < 0.001 |
| T1 | CV-2 | | 114 | IL-2 | | -0.36 (-0.59, -0.12) | | de | < 0.001 |
| T1 | CV-3 | | 47 | IL-2 | | -0.11 (-0.44, 0.21) | | c | < 0.001 |
| T1 | RA-1 | | 31 | IL-2 | | -0.22 (-0.60, 0.17) | | d | < 0.001 |
| T1 | RP-3 | | 93 | IL-2 | | -0.10 (-0.34, 0.13) | | c | < 0.001 |
| T1 | C-0 | | 85 | IL-4 | | 0.02 (-0.24, 0.28) | | c | < 0.001 |
| T1 | CV-2 | | 114 | IL-4 | | -0.13 (-0.36, 0.11) | | e | < 0.001 |
| T1 | CV-3 | | 47 | IL-4 | | 0.30 (-0.03, 0.62) | | bc | < 0.001 |
| T1 | RA-1 | | 31 | IL-4 | | 0.42 (0.04, 0.81) | | ab | < 0.001 |
| T1 | RP-3 | | 93 | IL-4 | | 0.20 (-0.04, 0.44) | | e | < 0.001 |
| T1 | C-0 | | 85 | IL-5 | | 0.66 (0.40, 0.92) | | a | < 0.001 |
| T1 | CV-2 | | 114 | IL-5 | | 0.49 (0.26, 0.72) | | ab | < 0.001 |
| T1 | CV-3 | | 47 | IL-5 | | 0.63 (0.30, 0.95) | | ab | 0.0016 |
| T1 | RA-1 | | 31 | IL-5 | | 0.65 (0.26, 1.03) | | ab | < 0.001 |
| T1 | RP-3 | | 93 | IL-5 | | 0.67 (0.43, 0.91) | | a | < 0.001 |
| T1 | C-0 | | 85 | IL-6 | | 1.25 (0.99, 1.51) | | b | < 0.001 |
| T1 | CV-2 | | 114 | IL-6 | | 0.85 (0.62, 1.09) | | c | < 0.001 |
| T1 | CV-3 | | 47 | IL-6 | | 0.80 (0.48, 1.13) | | ab | < 0.001 |
| T1 | RA-1 | | 31 | IL-6 | | 1.17 (0.78, 1.55) | | ce | < 0.001 |
| T1 | RP-3 | | 93 | IL-6 | | 1.10 (0.86, 1.33) | | b | < 0.001 |
| T1 | C-0 | | 85 | TNF-α | | -0.05 (-0.31, 0.21) | | c | < 0.001 |
| T1 | CV-2 | | 114 | TNF-α | | -0.40 (-0.64, -0.17) | | d | < 0.001 |
| T1 | CV-3 | | 47 | TNF-α | | 0.36 (0.04, 0.69) | | bc | 0.0016 |
| T1 | RA-1 | | 31 | TNF-α | | -0.64 (-1.03, -0.26) | | d | < 0.001 |
| T1 | RP-3 | | 93 | TNF-α | | -0.23 (-0.46, 0.01) | | c | < 0.001 |
| T2 | C-0 | | 18 | IFN-γ | | 0.73 (0.19, 1.28) | | b | 0.0222 |
| T2 | CV-3 | | 34 | IFN-γ | | 0.28 (-0.16, 0.72) | | ab | 0.0824 |
| T2 | RA-1 | | 17 | IFN-γ | | 0.55 (0.06, 1.04) | | b | < 0.001 |
| T2 | RP-3 | | 66 | IFN-γ | | 0.40 (0.10, 0.70) | | c | 0.0062 |
| T2 | C-0 | | 18 | IL-10 | | 0.32 (-0.23, 0.87) | | ab | 0.1942 |
| T2 | CV-3 | | 34 | IL-10 | | -0.04 (-0.48, 0.40) | | ab | 0.1618 |
| T2 | RA-1 | | 17 | IL-10 | | -0.01 (-0.50, 0.48) | | abc | 0.0773 |
| T2 | RP-3 | | 66 | IL-10 | | 0.06 (-0.24, 0.36) | | abc | 0.1311 |
| T2 | C-0 | | 18 | IL-13 | | -0.10 (-0.65, 0.45) | | a | 0.0222 |
| T2 | CV-3 | | 34 | IL-13 | | -0.30 (-0.74, 0.15) | | a | 0.0389 |
| T2 | RA-1 | | 17 | IL-13 | | -0.40 (-0.89, 0.09) | | ac | 0.0046 |
| T2 | RP-3 | | 66 | IL-13 | | -0.14 (-0.44, 0.16) | | ab | 0.0155 |
| T2 | C-0 | | 18 | IL-2 | | 0.04 (-0.51, 0.59) | | ab | 0.0508 |
| T2 | CV-3 | | 34 | IL-2 | | -0.19 (-0.63, 0.25) | | ab | 0.0824 |
| T2 | RA-1 | | 17 | IL-2 | | -0.20 (-0.69, 0.29) | | ac | 0.0212 |
| T2 | RP-3 | | 66 | IL-2 | | -0.06 (-0.36, 0.24) | | ab | 0.0335 |
| T2 | C-0 | | 18 | IL-4 | | -0.18 (-0.73, 0.36) | | a | 0.0229 |
| T2 | CV-3 | | 34 | IL-4 | | 0.18 (-0.26, 0.62) | | ab | 0.1618 |
| T2 | RA-1 | | 17 | IL-4 | | 0.27 (-0.22, 0.76) | | ab | 0.0114 |
| T2 | RP-3 | | 66 | IL-4 | | -0.12 (-0.42, 0.18) | | ab | 0.0174 |
| T2 | C-0 | | 18 | IL-5 | | 0.08 (-0.47, 0.63) | | ab | 0.0538 |
| T2 | CV-3 | | 34 | IL-5 | | -0.14 (-0.58, 0.30) | | ab | 0.0824 |
| T2 | RA-1 | | 17 | IL-5 | | 0.18 (-0.31, 0.67) | | ab | 0.0212 |
| T2 | RP-3 | | 66 | IL-5 | | -0.22 (-0.52, 0.08) | | a | 0.0062 |
| T2 | C-0 | | 18 | IL-6 | | 0.82 (0.27, 1.37) | | b | 0.0222 |
| T2 | CV-3 | | 34 | IL-6 | | 0.43 (-0.01, 0.88) | | b | 0.0389 |
| T2 | RA-1 | | 17 | IL-6 | | 0.62 (0.13, 1.11) | | b | < 0.001 |
| T2 | RP-3 | | 66 | IL-6 | | 0.26 (-0.04, 0.56) | | bc | 0.0251 |
| T2 | C-0 | | 18 | TNF-α | | 0.58 (0.03, 1.12) | | ab | 0.0538 |
| T2 | CV-3 | | 34 | TNF-α | | 0.03 (-0.42, 0.47) | | ab | 0.2214 |
| T2 | RA-1 | | 17 | TNF-α | | -0.63 (-1.12, -0.14) | | c | < 0.001 |
| T2 | RP-3 | | 66 | TNF-α | | 0.06 (-0.24, 0.36) | | abc | 0.1311 |
| Note: Panel A summarizes sample coverage for the cytokine-profile analysis by group × timepoint, where N denotes the number of subjects with complete cytokine data used in the mixed-effects model, and N_rows_used denotes the number of long-format rows contributed to the model. Panel B shows estimated marginal means (EMMs; log10 scale) with 95% CIs from linear mixed-effects models; within-group pairwise comparisons across cytokines were adjusted using the Benjamini–Hochberg FDR procedure. Shared CLD letters indicate no significant differences. For each cytokine, the *q* value is the smallest FDR-adjusted p value among all pairwise comparisons involving that cytokine within the same subset. This analysis compares relative cytokine levels within one group at one timepoint and does not evaluate longitudinal T1–T2 change. | | | | | | | | | |

**Supplementary Table 11. Covariate-adjusted associations between the RBD score and serum cytokines: regression coefficients and multiple-testing–adjusted results.**

| **Cytokine** | **Timepoint** | **β** | **95% CI** | ***P* value** | ***q* value** | **N** |
| --- | --- | --- | --- | --- | --- | --- |
| IL-2 | T1 | 0.175 | -0.466，0.815 | 0.589 | 0.933 | 85 |
|  | T2 | -0.008 | -0.167，0.151 | 0.919 | 0.919 | 125 |
| IL-4 | T1 | 0.025 | -0.526，0.575 | 0.929 | 0.933 | 85 |
|  | T2 | 0.011 | -0.163，0.186 | 0.899 | 0.919 | 125 |
| IL-5 | T1 | 0.521 | -0.010, 1.053 | 0.055 | 0.382 | 85 |
|  | T2 | -0.052 | -0.235, 0.132 | 0.578 | 0.919 | 125 |
| IL-6 | T1 | 0.14 | -0.497, 0.777 | 0.662 | 0.933 | 85 |
|  | T2 | -0.059 | -0.226, 0.108 | 0.486 | 0.919 | 125 |
| IL-10 | T1 | -0.076 | -0.642, 0.489 | 0.789 | 0.933 | 85 |
|  | T2 | -0.058 | -0.218, 0.103 | 0.478 | 0.919 | 125 |
| IL-13 | T1 | 0.483 | -0.087, 1.053 | 0.096 | 0.382 | 85 |
|  | T2 | 0.058 | -0.127, 0.244 | 0.533 | 0.919 | 125 |
| IFN-γ | T1 | 0.051 | -0.395, 0.497 | 0.82 | 0.933 | 85 |
|  | T2 | 0.026 | -0.156, 0.209 | 0.774 | 0.919 | 125 |
| TNF-α | T1 | 0.025 | -0.569, 0.619 | 0.933 | 0.933 | 85 |
|  | T2 | 0.023 | -0.152, 0.199 | 0.791 | 0.919 | 125 |
| Note: β denotes the standardized regression coefficient; 95% CI, 95% confidence interval. RBD_score was constructed as the mean of within-timepoint Z-scores of log10-transformed RBD-IgG against WT, Delta, and BA.5; cytokines were Z-scored based on log10-transformed values. T1 models adjusted for vaccine group, age, sex, and days since last vaccination; T2 models adjusted for vaccine group, age, sex, and the T1–T2 sampling interval (days). q values are Benjamini–Hochberg FDR-adjusted within each timepoint across 8 tests (m=8). N indicates the complete-case sample size. | | | | | | |

**Supplementary Table 12 . Summary of diagnostics for adjusted linear and linear mixed-effects models**

| **Model** | **Outcome** | **N** | **Adj. R²** | **Max VIF** | **BP test P** | **Shapiro P** | **Influential n** |
| --- | --- | --- | --- | --- | --- | --- | --- |
| **Panel A. Adjusted linear models** | | | | | | | |
| Cross-sectional LM | RBD_WT | 503 | 0.349 | 1.04 | <0.001 | <0.001 | 28 |
| Cross-sectional LM | RBD_Delta | 232 | 0.241 | 1.28 | <0.001 | 0.091 | 27 |
| Cross-sectional LM | RBD_BA.5 | 232 | 0.223 | 1.28 | <0.001 | 0.458 | 22 |
| Cross-sectional LM | IC50 | 163 | 0.084 | 1.27 | 0.238 | <0.001 | 7 |
| Cross-sectional LM | IL-5 | 505 | 0.003 | 1.04 | 0.870 | <0.001 | 42 |
| Cross-sectional LM | IL-13 | 505 | 0.006 | 1.04 | 0.811 | <0.001 | 14 |
| Cross-sectional LM | IL-2 | 505 | 0.059 | 1.04 | 0.017 | <0.001 | 37 |
| Cross-sectional LM | IL-6 | 505 | 0.014 | 1.04 | 0.383 | <0.001 | 42 |
| Cross-sectional LM | IL-10 | 505 | 0.026 | 1.04 | 0.208 | <0.001 | 29 |
| Cross-sectional LM | IFN-γ | 505 | -0.001 | 1.04 | 0.139 | <0.001 | 39 |
| Cross-sectional LM | TNF-α | 505 | 0.032 | 1.04 | 0.859 | <0.001 | 4 |
| Cross-sectional LM | IL-4 | 505 | 0.050 | 1.04 | 0.009 | <0.001 | 13 |
| T1 adjusted LM | RBD_WT | 285 | 0.298 | 2.23 | 0.161 | 0.077 | 14 |
| T1 adjusted LM | RBD_Delta | 85 | 0.812 | 6.87 | <0.001 | 0.009 | 9 |
| T1 adjusted LM | RBD_BA.5 | 85 | 0.741 | 6.87 | 0.002 | 0.719 | 9 |
| T1 adjusted LM | IC50 | 75 | 0.283 | 4.08 | 0.013 | 0.002 | 3 |
| T1 adjusted LM | IL-5 | 285 | -0.002 | 2.23 | 0.421 | <0.001 | 27 |
| T1 adjusted LM | IL-13 | 285 | 0.005 | 2.23 | 0.410 | <0.001 | 20 |
| T1 adjusted LM | IL-2 | 285 | 0.046 | 2.23 | 0.720 | <0.001 | 20 |
| T1 adjusted LM | IL-6 | 285 | -0.001 | 2.23 | 0.515 | <0.001 | 30 |
| T1 adjusted LM | IL-10 | 285 | 0.010 | 2.23 | 0.195 | <0.001 | 21 |
| T1 adjusted LM | IFN-γ | 285 | 0.008 | 2.23 | 0.117 | <0.001 | 22 |
| T1 adjusted LM | TNF-α | 285 | 0.025 | 2.23 | 0.177 | <0.001 | 3 |
| T1 adjusted LM | IL-4 | 285 | 0.059 | 2.23 | 0.387 | <0.001 | 12 |
| T2 adjusted LM | RBD_WT | 125 | 0.078 | 1.56 | 0.160 | <0.001 | 8 |
| T2 adjusted LM | RBD_Delta | 125 | 0.045 | 1.56 | 0.169 | <0.001 | 10 |
| T2 adjusted LM | RBD_BA.5 | 125 | 0.047 | 1.56 | 0.508 | <0.001 | 9 |
| T2 adjusted LM | IC50 | 66 | 0.144 | 1.78 | 0.127 | <0.001 | 10 |
| T2 adjusted LM | IL-5 | 125 | 0.013 | 1.56 | 0.046 | <0.001 | 7 |
| T2 adjusted LM | IL-13 | 125 | -0.003 | 1.56 | 0.219 | <0.001 | 4 |
| T2 adjusted LM | IL-2 | 125 | 0.011 | 1.56 | 0.711 | <0.001 | 7 |
| T2 adjusted LM | IL-6 | 125 | 0.137 | 1.56 | 0.113 | <0.001 | 7 |
| T2 adjusted LM | IL-10 | 125 | 0.052 | 1.56 | 0.697 | <0.001 | 9 |
| T2 adjusted LM | IFN-γ | 125 | 0.004 | 1.56 | 0.244 | <0.001 | 6 |
| T2 adjusted LM | TNF-α | 125 | 0.104 | 1.56 | 0.008 | <0.001 | 4 |
| T2 adjusted LM | IL-4 | 125 | 0.096 | 1.56 | 0.093 | <0.001 | 6 |
| **Panel B. Adjusted linear mixed-effects models** | | | | | | | |
| **Model** | **Outcome** | **N** | **Marginal R²** | **Conditional R²** | **Max VIF** | **Singular fit** | **Convergence message** |
| Main LMM | RBD_WT | 503 | 0.726 |  | 59.33 | Yes | boundary (singular) fit: see help('isSingular') |
| Main LMM | RBD_Delta | 232 | 0.639 |  | 10.56 | Yes | boundary (singular) fit: see help('isSingular') |
| Main LMM | RBD_BA.5 | 232 | 0.589 |  | 10.56 | Yes | boundary (singular) fit: see help('isSingular') |
| Main LMM | IC50 | 163 | 0.335 | 0.404 | 13.72 | No | None |
| Main LMM | IL-5 | 505 | 0.107 | 0.156 | 55.80 | No | None |
| Main LMM | IL-13 | 505 | 0.124 | 0.243 | 51.18 | No | None |
| Main LMM | IL-2 | 505 | 0.078 | 0.257 | 47.97 | No | None |
| Main LMM | IL-6 | 505 | 0.076 | 0.167 | 53.30 | No | None |
| Main LMM | IL-10 | 505 | 0.081 | 0.239 | 49.02 | No | None |
| Main LMM | IFN-γ | 505 | 0.019 | 0.163 | 50.52 | No | None |
| Main LMM | TNF-α | 505 | 0.057 | 0.197 | 50.46 | No | None |
| Main LMM | IL-4 | 505 | 0.066 | 0.279 | 46.57 | No | None |
| Interaction LMM | RBD_WT | 503 | 0.729 |  | 69.27 | Yes | boundary (singular) fit: see help('isSingular') |
| Interaction LMM | RBD_Delta | 232 | 0.640 |  | 13.96 | Yes | boundary (singular) fit: see help('isSingular') |
| Interaction LMM | RBD_BA.5 | 232 | 0.593 |  | 13.96 | Yes | boundary (singular) fit: see help('isSingular') |
| Interaction LMM | IC50 | 163 | 0.371 | 0.439 | 15.14 | No | None |
| Interaction LMM | IL-5 | 505 | 0.111 | 0.154 | 65.12 | No | None |
| Interaction LMM | IL-13 | 505 | 0.124 | 0.243 | 59.38 | No | None |
| Interaction LMM | IL-2 | 505 | 0.080 | 0.283 | 53.80 | No | None |
| Interaction LMM | IL-6 | 505 | 0.076 | 0.172 | 61.49 | No | None |
| Interaction LMM | IL-10 | 505 | 0.083 | 0.242 | 56.73 | No | None |
| Interaction LMM | IFN-γ | 505 | 0.020 | 0.167 | 58.40 | No | None |
| Interaction LMM | TNF-α | 505 | 0.057 | 0.195 | 58.68 | No | None |
| Interaction LMM | IL-4 | 505 | 0.066 | 0.281 | 53.35 | No | None |
| **Abbreviations:** BP, Breusch-Pagan; VIF, variance inflation factor. For linear models, diagnostics included adjusted R², multicollinearity, heteroscedasticity, residual normality, and influential observations. For mixed-effects models, marginal and conditional R², singularity, and convergence were additionally assessed. For mixed-effects models containing interaction terms, collinearity metrics were interpreted cautiously, with greater emphasis placed on convergence, singularity, and residual diagnostics. | | | | | | | |
